# Human sperm as an in vitro toxicity model: a versatile tool for assessing the risk of environmental contaminants

**DOI:** 10.1007/s00204-025-04035-x

**Published:** 2025-05-03

**Authors:** Shannen Keyser, Daniel Marcu, Morgan T. D. Davidse, Monique Bennett, Leslie Petrik, Liana Maree

**Affiliations:** 1 School of Nursing, Faculty of Community and Health Sciences, University of the Western Cape, Private Bag X17, Bellville, 7535 South Africa; 2 School of Biological Sciences, University of East Anglia, Norwhich, NR4 7TJ UK; 3 Comparative Spermatology Laboratory, Department of Medical Bioscience, University of the Western Cape, Private Bag X17, Bellville, 7535 South Africa; 4 Environmental and Nano Sciences Group, Department of Chemistry, University of the Western Cape, Private Bag X17, Bellville, 7535 South Africa

**Keywords:** Spermatozoa, In vitro model for toxicity screening, Alternative cellular models, Functional sperm parameters, CEC mixtures, Pesticides and pharmaceuticals

## Abstract

Contaminants of emerging concern (CECs) pose a significant threat to human and ecosystem health due to their persistence, bioaccumulation in higher trophic levels, and potential toxicity. While in vivo models are commonly used for toxicity screening, developing alternative in vitro techniques for rapid environmental risk assessment is essential. Spermatozoa, with their compartmentalized structure, measurable characteristics and sensitivity to environmental changes, offer potential as an in vitro model for toxicity screening. We evaluated the impact of selected CECs, including pharmaceuticals and pesticides, on sperm function in highly motile sperm subpopulations selected from donor semen. Standardised protocols were applied to assess various sperm functional parameters after 1–4 h of exposure to either individual or a mixture of chemicals. Our findings revealed that total motility is insufficient to detect subtle toxic effect. More responsive measures, such as sperm kinematics, induced hyperactivation, viability, mitochondrial membrane potential (MMP) and presence of reactive oxygen species (ROS) should be assessed to elucidate the effect of a toxic environment on sperm function. Most chemicals exerted a dose–response effect on sperm parameters, with the higher concentrations resulting in the most negative effects. The inherent sensitivity of human spermatozoa to oxidative stress, mitochondrial damage and energy metabolism, makes them a robust model for assessing toxicity. These features highlight their utility as an alternative cellular model for evaluating CECs and advancing risk assessment methodologies.

**Supplementary Information:**

The online version contains supplementary material available at 10.1007/s00204-025-04035-x.

## Introduction

With rising environmental pollution, assessing ecosystem health and its impact on organisms is crucial. Evaluation of such adverse effects typically involves the use of bioindicators and biomonitoring. Bioindicators monitor biodiversity and ecological changes (occurrence level) (Holt & Miller 2011), while biomonitoring makes use of biomarkers to measure physiological, genetic, or molecular responses to contaminants (quantitative response such as an increase or decrease) (Vallaeys et al. 2017). Toxicity screening further evaluates chemicals' toxic effects at cellular level (using organs, tissues or cells of target organisms) under a controlled environment (Madorran et al. 2020).

Many established in vivo and in vitro models are widely used in toxicity screening, but animal testing is costly, requires numerous specimens, and raises ethical concerns. With over 100 million vertebrates used annually in research, the European Union’s (EU) 3R strategy (Refinement, Reduction, Replacement) promotes alternative in vitro test systems that yield comparable results (Freires et al. 2017; Kollár et al. 2018). Similarly, the US National Academy of Science (U.S. NAS) and the US Environmental Protection Agency (U.S. EPA) support new approach methodologies (NAMs), including alternative organisms (e.g., zebrafish larvae, brine shrimp) and non-animal models (e.g., in silico or in vitro) that can inform chemical risk assessment and management of the ever-increasing number of new chemicals produced and used in commerce (U.S. NAS 2017; Madorran et al. 2020; U.S. EPA 2021; Kosnik et al. 2022; Escher et al. 2023; Freires et al. 2023). Thus, the development and validation of new in vitro techniques for environmental risk assessment should also be prioritised (Shaliutina et al. 2021). In vitro models are cost-effective, standardized, reproducible, and enable high-throughput testing (Jain et al. 2018; Shaliutina et al. 2021), offering better control and insights into chemical toxicity mechanisms (Jain et al. 2018).

Spermatozoa are specialized cells suitable for in vitro toxicology models due to their inherent compartmentalized structure and measurable traits. Spermatozoa typically consist of a head (acrosome and DNA), neck (centrioles), and flagellum (midpiece with mitochondria arranged in a helix around the axoneme and principal piece with protein fibers such as the outer dense fibers, microtubules and motor proteins) (Mortimer 2018). These male gametes perform essential functions, such as movement, cell recognition, secretion and membrane fusion to fertilize oocytes (Aitken 1990). With limited DNA repair and antioxidant defences (Moretti et al. 2023), spermatozoa rely on external signals, making them responsive to the female reproductive environment (Ghersevich et al. 2015; Sysoeva et al. 2022), but also vulnerable to environmentally-induced damage (Shaliutina et al. 2021).

Spermatozoa's structural and functional traits allow for measuring various endpoints, such as vitality, motility, acrosome reaction, MMP intactness, ROS levels and DNA fragmentation, making them valuable in toxicity screening (Moretti et al. 2023). Standardized protocols, like those endorsed by the World Health Organization (WHO) for human spermatozoa (WHO 2021; Björndahl et al. 2022), ensure reliable and reproducible assessments. Sperm cells are also abundant, inexpensive to produce, and easy to obtain from semen donors. Studies on spermatozoa as in vitro models across various phyla (Marcu et al. 2023) have demonstrated their dose-dependent responses to environmental contaminants, highlighting their potential as in vitro models.

Contaminants of emerging concern (CECs) are chemicals or materials, either natural or synthetic, that are present in/or polluting the environment and may significantly affect metabolism and health (Sauvé & Desrosiers 2014). These include pharmaceuticals and personal care products (PPCPs), pesticides and industrial chemicals like flame retardants and plasticizers (Marcu et al. 2023). Over the past three decades, attention to CECs has grown due to their harmful impacts on human and ecosystem health, leading to increased efforts in registration, monitoring, and ecological screening (Diamond et al. 2011; Naidu et al. 2021). Their persistence, bioaccumulation, and potential for synergistic toxic effects make toxicity screening a critical priority (Kortenkamp & Faust 2018; Kassotis & Stapleton 2019; Neale et al. 2020). Several studies have explored the use of spermatozoa as a sensitive model for toxicological assessments such as investigating the toxic effects of bisphenol A (BPA) and phthalates (Kotwicka et al. 2016; Castellini et al. 2021), heavy metals (Jeng 2014; López-Botella et al. 2021) and pesticides (Moreira et al. 2021; Chiu et al. 2022). Despite these findings, there remains a gap in the literature regarding the application of spermatozoa as a screening tool for broader categories of CECs, particularly in relation to complex mixtures and long-term exposure scenarios.

In this study, we investigated the use of human spermatozoa as a potential model for toxicity screening, as previously recommended in studies evaluating in vivo toxins and antioxidant supplementation (Vollmer et al. 2019; Moretti et al. 2023). Human spermatozoa were exposed to selected CECs (three pharmaceuticals and two pesticides) at various concentrations to determine: 1) which sperm characteristics are most sensitive as an endpoint, 2) which exposure level has the most severe impact on measured endpoints, and 3) possible mechanisms of action of the contaminants. To verify the effects of CECs on sperm function, we subsequently used a mixture of the five CECs at environmentally-relevant concentrations to reveal the versatility of human spermatozoa as a screening tool.

## Methods and Materials

### Media Preparation

All chemicals for media preparation were sourced from Sigma-Aldrich (Cape Town, South Africa) and of analytical grade or MQ 100 standard. The five contaminants selected for this study—naproxen (N8280, ≥ 98.5% purity), diclofenac (D6899, ≥ 98.0% purity), sulfamethoxazole (S7507, ≥ 98.0% purity), atrazine (49,085, ≥ 99.0% purity), and chlorpyrifos (45,395, ≥ 98.0% purity)—were chosen based on their prevalence in False Bay's near-shore marine environment (Petrik et al. 2017; Ojemaye et al. 2020, 2021; Ojemaye & Petrik 2022). The chemicals were prepared as stock solutions in 100% dimethyl sulfoxide (DMSO, ≥ 99.5% purity) and diluted in human tubal fluid (HTF), with all media excluding 1% human serum albumin (HSA) to only assess the chemicals' direct impact on sperm function and toxicity. The working solutions for individual chemical exposures contained no more than 1% DMSO, while the chemical mixtures contained no more than 0.03% DMSO.

For single chemical exposures, we intentionally used concentrations higher than what has been reported in the environment to assess the toxicity thresholds for human spermatozoa and to validate spermatozoa as a potential model for assessing CECs. Firstly, it was imperative to demonstrate that spermatozoa exhibit characteristic toxicological responses and identify the most reliable test parameters for toxicity evaluation. The highest concentrations of each chemical were selected upon a reduction of at least 15% in total motility and in vitality compared to the control (Gruber et al. 2020). Lower concentrations for dose–response assessments were selected by serial dilution (halving the highest concentration). This stepwise approach allowed us to establish clear toxicity effects, optimize test conditions, and subsequently assess the combined effects of chemicals at lower, environmentally-relevant concentrations, reflecting real-world exposure scenarios.

#### Controls

HTF was prepared as both non-capacitating and capacitating media, serving as negative and positive controls, respectively. Non-capacitating HTF included 1.4845 g NaCl, 0.0875 g KCl, 0.024 g MgSO_4_, 0.0125 g KH_2_PO_4_, 0.525 g NaHCO_3_, 0.009 g Na pyruvate, 0.12525 g glucose, 0.0075 g phenol red, and 0.784 g Na lactate. Capacitating HTF (CAP) was made by adding 0.105 g NaHCO_3_, 1.1915 g HEPES, and 0.6 mL NaOH to the non-capacitating medium (Mortimer 1994).

#### Single Chemical Exposure

Naproxen (NPX) stock solution (100 mg/mL) was diluted to working concentrations of 0.125 mg/mL, 0.250 mg/mL, 0.500 mg/mL, and 1.0 mg/mL in HTF. Diclofenac (DCF) stock solution (35 mg/mL) was diluted to 0.032 mg/mL, 0.06 mg/mL, 0.125 mg/mL, and 0.25 mg/mL in HTF. Sulfamethoxazole (SX) stock solution (50 mg/mL) was diluted to 0.062 mg/mL, 0.125 mg/mL, 0.250 mg/mL, and 0.500 mg/mL in HTF. Atrazine (ATZ) and Chlorpyrifos (CHL) stock solutions (100 mg/mL each) were diluted to 300 µM, 600 µM, 1200 µM, and 2400 µM in HTF. These concentrations are denoted as T1, T2, T3, and T4 in the results, representing increasing chemical concentrations.

#### Collective Mixture Exposure

Three collective mixtures of the five chemicals were prepared in HTF at increasing concentrations (ng/L; µg/L; µg/mL). The first mixture (MIX1) contained all five chemicals at environmentally relevant concentrations: 4 ng/L DCF, 2.1 ng/L NPX, 5 ng/L SX, 4.8 ng/L CHL, and 2 ng/L ATZ (Petrik et al. 2017; Ojemaye et al. 2020, 2021; Ojemaye & Petrik 2022). The second mixture (MIX2) had concentrations of 4 µg/L DCF, 2.1 µg/L NPX, 5 µg/L SX, 4.8 µg/L CHL, and 2 µg/L ATZ. The third mixture (MIX3) contained 4 µg/mL DCF, 2.1 µg/mL NPX, 5 µg/mL SX, 4.8 µg/mL CHL, and 2 µg/mL ATZ.

### Sample collection and standard semen analysis

Semen samples were collected through masturbation after two to three days of sexual abstinence as part of a donor program (Comparative Spermatology Laboratory, Department of Medical Bioscience, University of the Western Cape). The samples were incubated at 37°C in a 5% CO_2_ incubator for 30–60 min to liquefy before processing for standard semen analysis as per the World Health Organization Laboratory Manual (WHO 2021). Semen volume, pH, total motility, progressive motility, sperm concentration, and mucus penetration were assessed (see Table S1). A minimum total sperm motility of 20% in semen was used as a cut-off for sample selection (WHO 2021).

### Sample Preparation

All media, stains and slides were preheated to 37°C before use. Semen samples were separated using the V-GRAD 80/40 double density gradient centrifugation technique (Delfran, Johannesburg, South Africa). Equal volumes (300 µL) of preheated V-GRAD 80%, V-GRAD 40%, and semen were pipetted into 2 mL Eppendorf tubes, which were centrifuged at room temperature (RT) at 500 *g* for 15 min to separate viable, motile spermatozoa from non-motile spermatozoa, seminal plasma, and debris. The high motility sperm pellet was resuspended in 300 µL V-SPERM Wash and centrifuged at 500 *g* (RT) for 10 min. The washed pellets were re-suspended in various media for treatment, and sperm concentrations were adjusted to 15–25 × 10^6^/mL before further analysis.

### Sperm motility, kinematics, mucus penetration and concentration

Sperm parameters were assessed using the Motility module of the Sperm Class Analyzer (SCA®) computer-aided sperm analysis (CASA) system (Microptic S.L., Barcelona, Spain), version 6.2. A Basler acA1300-200uc digital camera (Microptic S.L., Barcelona, Spain) attached to a Nikon Eclipse 50i microscope (IMP, Cape Town, South Africa) with a heated stage and positive phase optics was used. A four chamber, 20 μm-depth Leja slide (Leja Products B.V., The Netherlands) was loaded with 3 µL of semen and placed on the heated stage. Two fields were captured at 50 frames per second (f/s), analysing a minimum of 200 motile sperm per sample using a 10 × objective.

During semen analysis, total sperm motility (%), progressive motility (%), mucus penetration (× 10^6^/mL), and concentration (× 10^6^/mL) were assessed using SCA® software settings for human sperm. Mucus penetration was determined using kinematic cut-off values: average path velocity (VAP) > 25 µm/s, straightness (STR) > 80%, and amplitude of lateral head displacement (ALH) between 2.5 and 7.5 µm.

Motility and kinematics were assessed at 5, 30, and 60-min intervals after sperm preparations were exposed to the various chemical concentrations and/or mixtures. A preheated 20 μm-depth, eight chamber Leja slide was loaded with 2 µL of sperm preparation, placed on the heated stage, and sperm motility parameters were analysed at 50 f/s. Parameters included total motility, progressive motility, swimming speeds (rapid, medium, and slow), curvilinear velocity (VCL), VAP, straight-line velocity (VSL), STR, linearity (LIN), wobble (WOB), ALH, and beat cross frequency (BCF). ALH was measured as half the width of the VCL track, rather than the full VCL wave or double riser value as described by Mortimer (1994, 2013).

### Hyperactivation

Hyperactivation was analysed using the Motility module of SCA® with human spermatozoa configuration settings. Sperm preparations were incubated in HTF for 5 min for acclimation before analysis. Highly motile sperm in HTF were flushed with selected individual chemical treatments as previously described. The flush technique, based on Boshoff et al. (2018), involved loading 0.5 µL of motile sperm preparation into each chamber of a preheated 20 μm-depth eight-chamber Leja slide, followed by 1.5 µL of preheated treatment. Hyperactivation percentage was assessed using cut-off values: VCL > 150 µm/s, LIN < 50%, and ALH > 7 µm (3.5 for SCA®) for at least 200 motile spermatozoa after 5, 15, 30, 45, and 60 min of incubation. Analysis was done at 50 f/s using positive phase contrast optics, a green filter, and a 10 × objective.

### Sperm Vitality

Sperm preparations were incubated for 60 min in HTF in addition to the highest concentration of each individual chemical and all three collective mixtures. After incubation, samples were stained for vitality using BrightVit (Microptic S.L., Barcelona, Spain), an eosin-nigrosin stain. The samples were stained at a ratio of 1:4 (10 µL sample to 40 µL stain) for 10–15 min at 37°C. Vitality smears (20 µL) were prepared following Microptic S.L. (2022) guidelines, air-dried, and mounted with DPX mounting medium (Sigma Aldrich, Cape Town, South Africa). Stained smears were viewed using brightfield optics and a 40 × objective on a Nikon Eclipse 50i microscope. The percentage of live (unstained, white) and dead (pink-stained) spermatozoa was calculated after manually assessing at least 200 sperm per slide.

### Sperm viability

The Cell Proliferation Reagent WST-1 assay (Cat. No. 11 644 807 001, Sigma-Aldrich, Cape Town, South Africa) was used to assess cell viability of sperm subpopulations after exposure to individual DCF concentrations and the three collective mixtures. Sperm subpopulations were incubated at 37°C for up to 4 h with WST-1 stain, after which absorbance was measured to determine viability. Three replicates and a blank (HTF with WST-1 stain, without spermatozoa) were included for each treatment. Samples were prepared by mixing 360 µL of sperm sample with 40 µL of WST-1 stain in an Eppendorf tube at a 9:1 ratio. Then, 100 µL of the mixture was pipetted into a 96-well plate (three wells per treatment). The plate was analysed for the baseline measurement and again after 4 h of incubation using an ELISA reader (Microplate reader, Multiskan EX, Thermo Scientific) set to 450 nm absorbance with a reference wavelength of 650 nm. Viability was normalized to the control (HTF).

### Reactive Oxygen Species

Sperm preparations were incubated at 37°C for 60 min in the three collective mixtures. After incubation, 180 µL of sperm preparation was stained with 20 µM Dihydroethidium (DHE; excitation = 518 nm, emission = 605 nm; Molecular Probes, Eugene, OR, USA) in the dark at 37°C for 15 min to detect spermatozoa positive for ROS. The stained sperm were centrifuged at 500 *g* for 5 min at RT, and the pellets were re-suspended in the chemical mixtures. After resuspension, 5–10 µL of the suspension was placed on a clean slide with a coverslip and immediately analysed using a Nikon Eclipse 50i fluorescence microscope with a 100 × oil immersion objective and a triband filter (MXU440, excitation wavelengths: 457 nm = blue, 530 nm = green, and 628 nm = red). The percentage of spermatozoa positive for ROS was manually calculated from at least 100 spermatozoa assessed per treatment.

### Mitochondrial Membrane Potential

Prior to the start of this investigation, the Sigma Aldrich mitochondrial membrane potential (MMP) protocol kit (CS0390) was optimized for volume and cell concentration to best suited for the assessment of human spermatozoa. Sperm preparations were incubated at 37°C for 60 min in the four concentrations of DCF and the three collective mixtures. Following incubation, the exposed sperm preparations were stained at a ratio of 1:1 in the dark at 37°C for 20 min in suspension (200 µL) with the MMP staining solution, which consisted of 160 µL dH_2_O, 40 µL JC-5 buffer, and 1 µL of the frozen MMP 200 × stock solution. After incubation, the suspensions were centrifuged at 500 *g* for 5 min at 5–7°C, and the pellets were washed before being re-suspended in 200 µL JC-1 buffer (prepared by mixing 80 µL JC-5 buffer with 320 µL dH_2_O) and cooled on ice. The suspensions were centrifuged again as described, and the pellets were re-suspended in the remaining 200 µL of JC-1 buffer. A single drop (5–10 µL) of the suspension was placed on a clean slide, covered with a coverslip, and immediately analysed using the same equipment described for ROS analysis. The percentage of spermatozoa with intact MMP was calculated after manually assessing at least 100 spermatozoa per treatment.

#### Statistical analysis

Data collection, storage, and graphical analysis were performed using Microsoft Office Excel^™^ 2016 (Microsoft Corporation, Redmond, Washington, United States). Basic summary statistics were calculated using MedCalc statistical software version 14.8.1 (Mariakerke, Gent, Belgium), and results were expressed as mean ± standard deviation in all tables. The distribution of the data was first assessed using the Shapiro–Wilk test. For normally distributed data, the Student’s t-test was used, while for non-normally distributed data, the Mann–Whitney test was applied. One-way analysis of variance (ANOVA) for parametric data or the Kruskal–Wallis test for non-parametric data were used where applicable to compare treatments. A significance level of *p* < 0.05 was considered statistically significant. Outlier detection was determined based on the Tukey test.

R software version 4.0.1 was used to generate density plots, box plots, and modified scatter plots. Scatter plots visually display individual data points for a specific variable (such as total motility, progressive motility, VCL, VAP, and hyperactivation) across different time intervals.

## Results

### Responsiveness of Spermatozoa

**Fig. 1:**
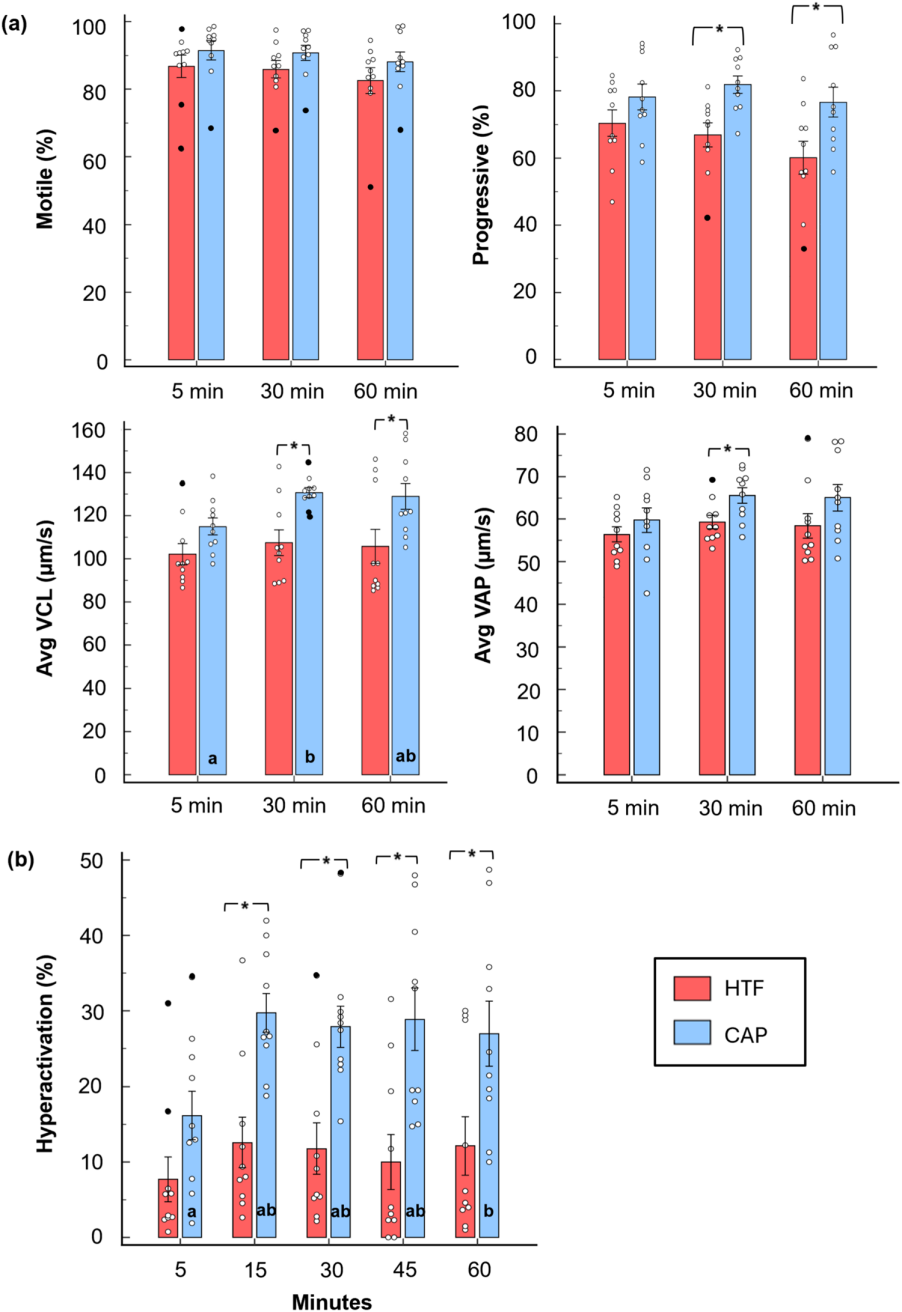
Spermatozoa’s responsiveness to exposure with capacitating HTF medium as compared to non-capacitating HTF medium. The effects of capacitating HTF medium on sperm motility (percentage motile and progressive) and kinematic parameters (VLC and VAP) (**a**) in addition to the percentage sperm hyperactivation over 60 (**b**) Note: ^a,b^ One-way ANOVA for parametric distributions or Kruskal-Wallis test for non-parametric distributions was used to calculate significant (p > 0.05) differences between time intervals for individual media. Student’s t-test or the Mann––Whitney test when normal distribution was void, was used to calculate significant difference between the media for an individual time point and is indicated by an asterisk (*). Data are presented as means ± SEM, n = 10, black dots indicate detected outliers. *Avg* average, *CAP* capacitating, *HTF* human tubal fluid (control), *min* minutes, *SEM* standard error of the mean, *VAP* average path velocity, *VCL* curvilinear velocity

To evaluate human spermatozoa's responsiveness to external stimuli over time, CAP medium (positive control) was compared with HTF (negative control) for assessing motility parameters and inducing hyperactivation over a 60-min period (Fig. 1a & 1b, Table S2 & S3). As shown in Fig. 1a, average values of samples exposed to CAP predominantly exceeded those of samples incubated in HTF across all four motility parameters. The presence of outliers among the individual data points highlights the heterogeneity within human sperm subpopulations, despite the use of highly motile spermatozoa, which were separated from the semen for exposure. It is important to note that HTF maintained relatively consistent sperm characteristics throughout the incubation period, whereas the presence of an external stimulus such as CAP resulted in noticeable alterations to these sperm characteristics over time.

Among the four selected sperm motility parameters, total motility showed no significant differences between HTF and CAP at any time point. However, CAP treatment significantly increased progressive motility, VCL and VAP. Progressive motility was higher in CAP-treated samples at both 30 min (81.9 ± 8.0%) and 60 min (76.6 ± 14.1%) compared to HTF-treated samples (30 min: 66.9 ± 11.3%; 60 min: 60.1 ± 15.6%). Similarly, VCL increased significantly with CAP at 5 min (115.0 ± 12.3 µm/s) and 30 min (130.6 ± 7.2 µm/s), a trend not observed in HTF-treated samples. In contrast to VCL, significant differences in VAP were observed only between media, rather than across time points. Specifically, HTF-treated samples (59.3 ± 4.9 µm/s) exhibited significantly lower VAP values at 30 min compared to CAP-treated samples (65.5 ± 5.8 µm/s).

Over the 60-min period (see Table S4 & Fig. 1b), CAP treatment significantly increased sperm hyperactivation, with values on average 2.1 to 2.9 times higher than in HTF-treated samples, across all time points. HTF-treated samples exhibited stable hyperactivation percentages throughout the incubation period, with averages ranging from 7.7% to 12.9%. In contrast, CAP-treated samples demonstrated time-dependent variation, with hyperactivation peaking between 15 and 45 min. This observation underscores the importance of selecting the appropriate incubation period and sperm motility parameters when assessing the effects of environmental changes on sperm functionality.

### Effect of Individual Chemicals on Sperm Parameters

**Fig. 2:**
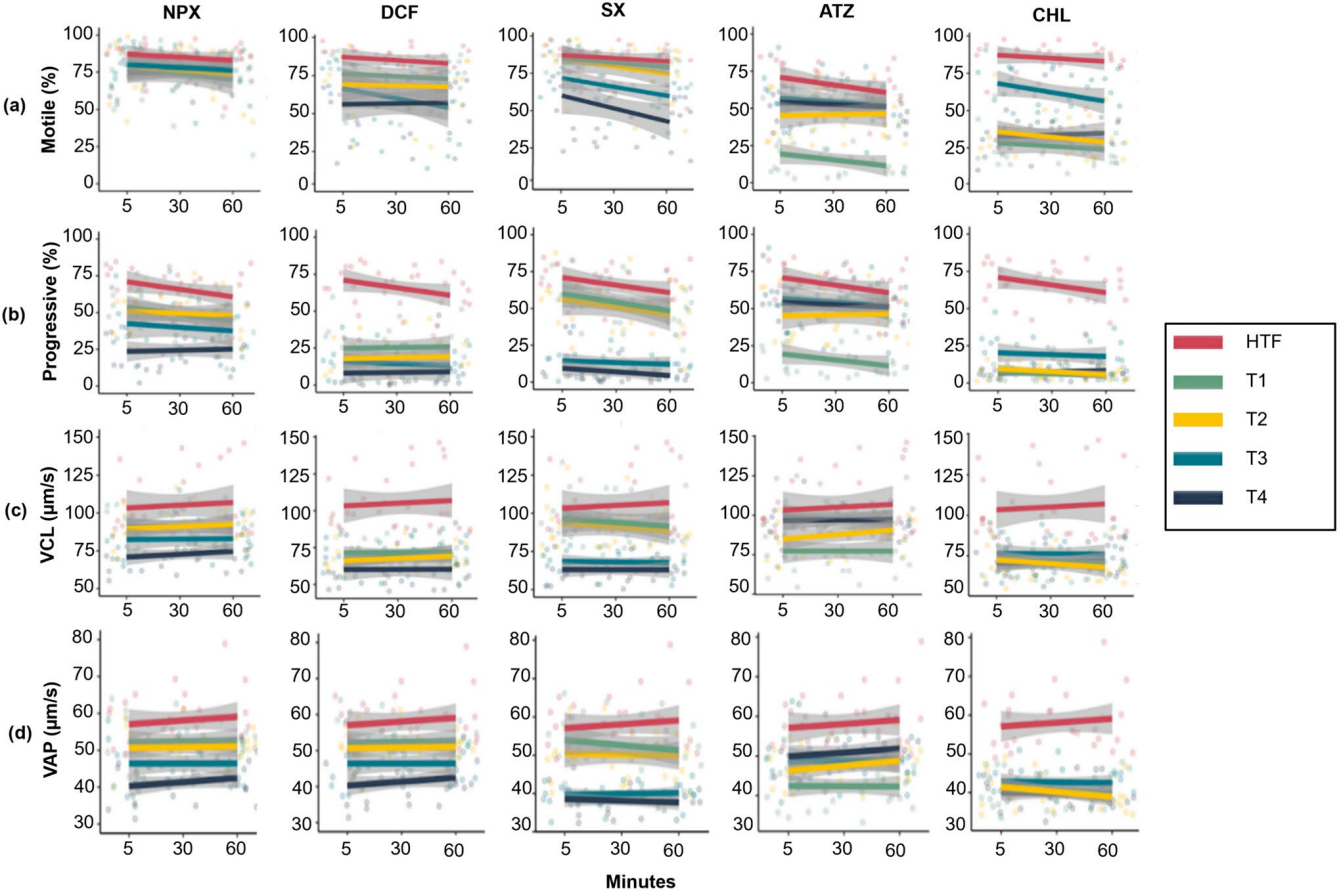
Effects of incerasing conentrations of naproxen, diclofenac, sulfamethoxazole, atrazine and chlorpyrifos on percentage total sperm motility (**a**), percentage progressive motility (**b**), average VCL (**c**) and average VAP (**d**) kinematic parameters over 60 minutes exposure. Note: T1-T4 is equal to the ascending concentrations of each individual treatment. Faded dots on the scatter plot represent individual data points, whereas the line indicates a trendline. The 95% confidence interval are displayed as the grey shadow around the trendline, n=10. Actual averages and standard deviations are portrayed in Table S2 and S3. *ATZ* atrazine, *CHL* chlorpyrifos, *CTRL* HTF control, *DCF* diclofenac, *NPX* naproxen, *SX* sulfamethoxazole, *VAP* average path velocity, *VCL* curvilinear velocity

**Fig. 3:**
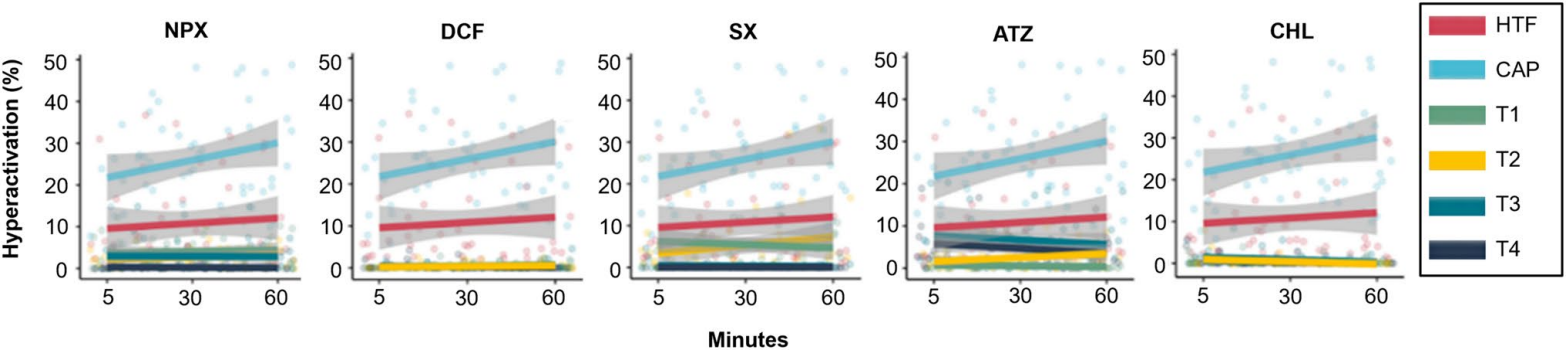
The effects of increasing concentration levels of naproxen, diclofenac, sulfamethoxazole, atrazine and chlorpyrifos on percentage sperm hyeractivation over 60 minutes exposure. Note: Note: T1-T4 is equal to the ascending concentrations of each individual treatment. Faded dots on the scatter plot represent individual data points, whereas the line indicates a trendline. The 95% confidence interval are displayed as the grey shadow around the trendline, n=10. Actual averages and standard deviations are portrayed in Table S4. *ATZ* atrazine, *CAP* capacitating HTF, *CHL* chlorpyrifos, *CTRL* HTF control, *DCF* diclofenac, *NPX* naproxen, *SX* sulfamethoxazole, *T* treatment

**Fig. 4:**
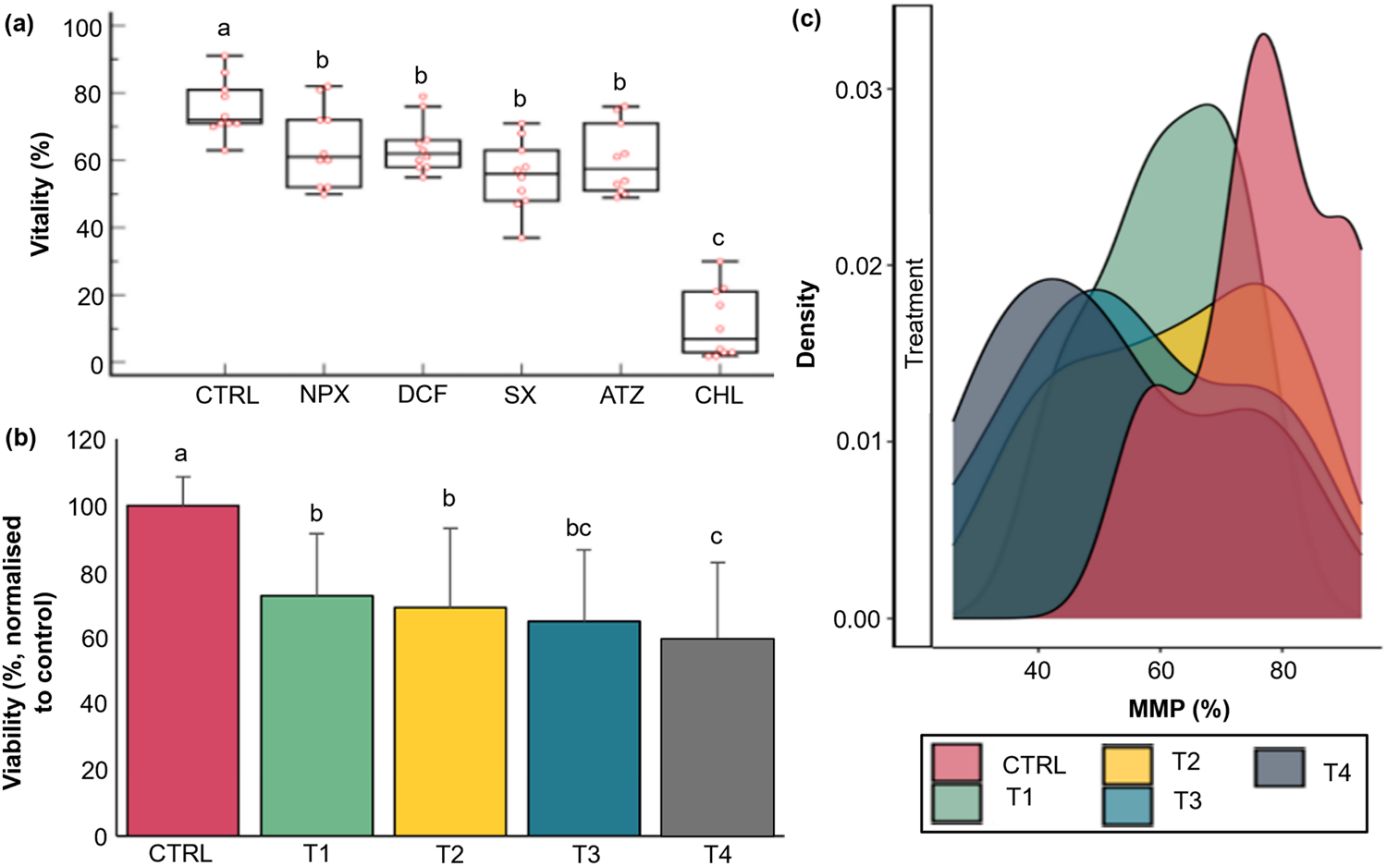
Effects of chemical exposure on percentage sperm vitality, viability and mitochondrial membrane potential. Effects of naproxen, diclofenac, sulfamethoxazole, atrazine and chlorpyrifos on percentage sperm vitality after 60 minutes exposure to the highest concentrations (T4) (**a**). Effect of increasing concentrations (T1-T4) of diclofenac on percentage sperm viability (normalized to the the untreated control) after 4 hours incubation (mean ± SD) (**b**), and sperm mitochondrial memebrane potential after 60 minutes exposure (**c**). Note: The box plots (**a**) provide a visual depiction of the variability present within a dataset by presenting the median, upper and lower quartiles, as well as the minimum and maximum values and outlier. ^a, b, c^ One-way ANOVA for parametric distributions or Kruskal-Wallis test for non-parametric distributions was used to calculate significatnt (p< 0.05) differences between approximation of the underlying data distribution. The peak of a density plot reveals regions of where data is concerntrated in a specific domain (**c**). T1-T4 are equal to ascending concentrations of each diclofenac treatment, n-10. *ATZ* atrazine, *CHL* chlorpyrifos, *CTRL* HTF control, *DCF* diclofenac, *MMP* mithochondrial membrane potential, *NPX* naproxen, *SD* standard deviation, *SX* sulfamethoxazole

To further evaluate the potential of human spermatozoa as a toxicology model, spermatozoa were exposed to a range of relatively high concentrations of NPX, DCF, SX, ATZ, and CHL (see Figs. 2, 3, 4). This approach was used to determine whether spermatozoa exhibit measurable toxicological responses, establish baseline effects, and identify the most suitable test parameters before assessing environmentally-relevant concentrations and chemical mixtures. Throughout the 60-min exposure period, various chemicals negatively impacted sperm functional parameters, as demonstrated in Tables S2-S4. However, the results also reveal the differential effects of these chemicals on individual sperm parameters, emphasizing the varying sensitivities of sperm characteristics to different concentrations and types of compounds.

#### Motility, Kinematics and Hyperactivation

The separation of the trendlines and the faded dots in Fig. 2a-d indicate significant differences between HTF and the dose-dependent effects of the four selected chemical concentrations on most sperm parameters over time. NPX did not significantly affect total motility, while all other chemicals significantly reduced motility compared to the HTF control (5 min: 86.8 ± 10.4%; 30 min: 85.9 ± 8.1%; 60 min: 82.6 ± 12.0%) across the three-time intervals (see Table S2 & Fig. 2a). Specifically, DCF significantly decreased total motility at the three highest concentrations (T2, T3, T4) at 5 min, with reductions observed at all concentrations (T1, T2, T3, T4) at 30 and 60 min. SX caused significant reductions at T3 and T4 across all three time points, with T4 (43.3 ± 15.1%) showing a stronger effect than T3 (61.0 ± 13.3%) at 60 min. For ATZ, a significant reduction in total motility was observed only at the lowest concentration (T1) at all time points, with values decreasing at 5 min (51.4 ± 18.4%), 30 min (40.2 ± 14.3%), and 60 min (36.1 ± 16.5%). CHL significantly decreased total motility at all time points and concentrations (T1, T2, T3, T4), with T3 (5 min: 70.2 ± 12.7%; 30 min: 58.3 ± 15.3%; 60 min: 58.3 ± 15.3%) showing the least negative impact.

All chemicals significantly decreased the percentage of progressive motility compared to the HTF control (5 min: 70.4 ± 12.4%; 30 min: 66.9 ± 11.3%; 60 min: 60.1 ± 15.6%) across all three time points (Fig. 2b). For NPX, the highest concentration (T4) caused the most significant reduction in progressive motility, with values dropping to 22.8 ± 13.3% at 5 min, 25.9 ± 11.7% at 30 min, and 24.4 ± 10.4% at 60 min. DCF showed a greater reduction in progressive motility at T4 (9.1 ± 12.6%) compared to T2 (18.3 ± 11.4%) at 5 min. At 30 min, T4 (6.2 ± 8.7%) was significantly lower than T1 (25.2 ± 10.3%), T2 (17.7 ± 11.8%), and T3 (16.0 ± 10.6%). SX caused a reduction in progressive motility, with a clear distinction between the highest (T3 and T4) and lowest (T1 and T2) concentrations, showing the most significant reductions at T3 and T4. For ATZ, the lowest concentration (T1) significantly reduced progressive motility at all time points, with values at 5 min (19.4 ± 16.2%), 30 min (15.6 ± 10.8%), and 60 min (11.4 ± 7.3%) significantly lower than HTF and higher concentrations. T3 (55.1 ± 17.7%) only significantly reduced progressive motility at 5 min. For CHL, T3 (5 min: 22.0 ± 13.3%; 60 min: 19.6 ± 10.6%) had the least negative impact on progressive motility at the 5- and 60-min intervals compared to other concentrations.

Swimming speed analysis revealed a general reduction in both VCL and VAP with increasing concentrations of all chemicals compared to the HTF control (Table S3 & Fig. 2c-d). The reductions were concentration- and time-dependent for NPX, SX, and AZT (see Table S3). For SX, there was a clear separation between higher concentrations (T3, T4) and lower concentrations (T1, T2) across all time points, as seen in Fig. 2c and 2d. This suggests that higher concentrations of SX had a more significant effect on swimming speed compared to the lower concentrations. On the other hand, AZT only significantly reduced VCL at the lowest concentrations, namely T1 (5 min: 76.9 ± 15.1 µm/s; 30 min: 78.3 ± 14.8 µm/s; 60 min: 76.8 ± 9.3 µm/s) and T2 (30 min: 88.8 ± 12.7 µm/s). Similarly, VAP was significantly lower at only the lowest concentration of ATZ, T1 (5 min: 42.3 ± 5.4 µm/s; 30 min: 42.5 ± 5.2 µm/s; 60 min: 42.2 ± 3.4 µm/s). CHL caused a significant reduction in both VCL and VAP at all concentrations and time points, as seen by the distinct separation between HTF and CHL-treated samples in Fig. 2c and 2d. This highlights CHL's consistent and pronounced effect on swimming speed across all concentrations and time intervals.

As previously noted, human spermatozoa incubated in HTF maintained consistent hyperactivation percentages over the 60-min period. In contrast, CAP-treated samples showed significantly higher hyperactivation percentages at four of the five time points (see Fig. 3 & Table S4). This further supports CAP’s stimulatory effect on sperm motility, specifically enhancing hyperactivation compared to the HTF control. Exposure to NPX, DCF and CHL significantly reduced hyperactivation percentages across all concentrations, particularly during the early time points (5–30 min). At later intervals (45–60 min), a dose-dependent effect emerged, with higher chemical concentrations (T3 and T4) causing greater reduction compared to lower concentrations (T1 and T2). For SX, only T3 and T4 significantly reduced hyperactivation at the initial time points, but over time T1 and T2 also showed significant negative effects. ATZ showed a distinct pattern with lower concentrations (T1, T2) causing significant reduction in hyperactivation during the initial 5–15 min. After 30 min, varied effects were observed for T3 and T4, whereas T1 and T2 consistently displaying significantly greater effects throughout the duration of the experiment.

#### Vitality, Viability and Mitochondrial Membrane Potential

Displayed in Fig. 4 are the effects of chemical exposure on sperm vitality (Fig. 4a), viability (Fig. 4b) and MMP intactness (Fig. 4c). After 60 min, T4 treatments significantly decreased vitality percentages compared to the HTF control. NPX (64.3 ± 11.8%), DCF (64.1 ± 7.8%), SX (55.5 ± 10.3%), and ATZ (60.2 ± 10.5%) had similar vitality percentages, while CHL (11.4 ± 10.3%) caused the most severe decline. After 4 h, DCF significantly reduced sperm viability at higher concentrations, with T4 (59.5 ± 23.2%) showing the greatest reduction. DCF also significantly impaired MMP (Fig. 4c), particularly at T2 (62.3 ± 17.1%), T3 (55.9 ± 18.5%), and T4 (52.4 ± 18.5%) compared to the control (77.5 ± 12.3%).

### Validation of Sperm Toxicology Model

**Fig. 5:**
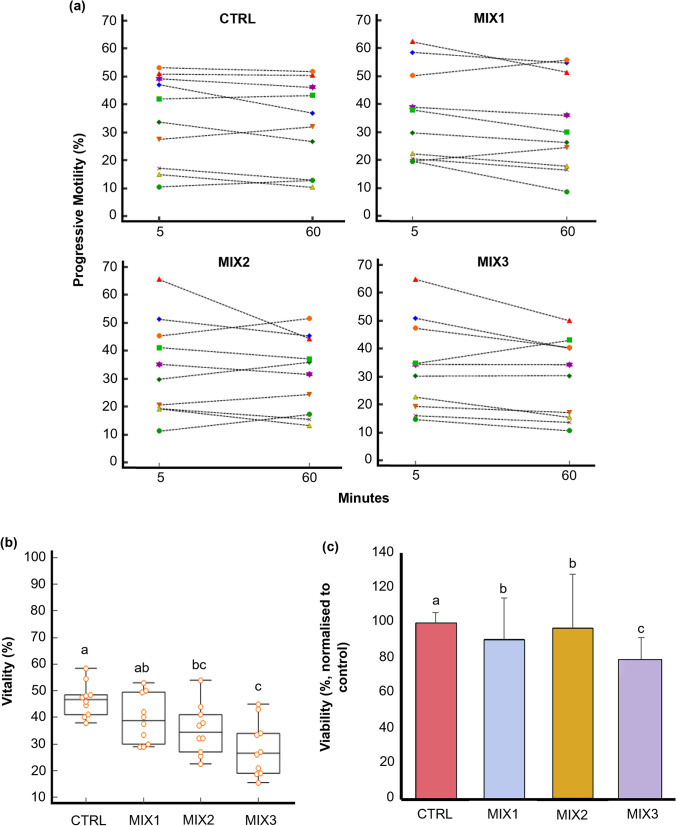

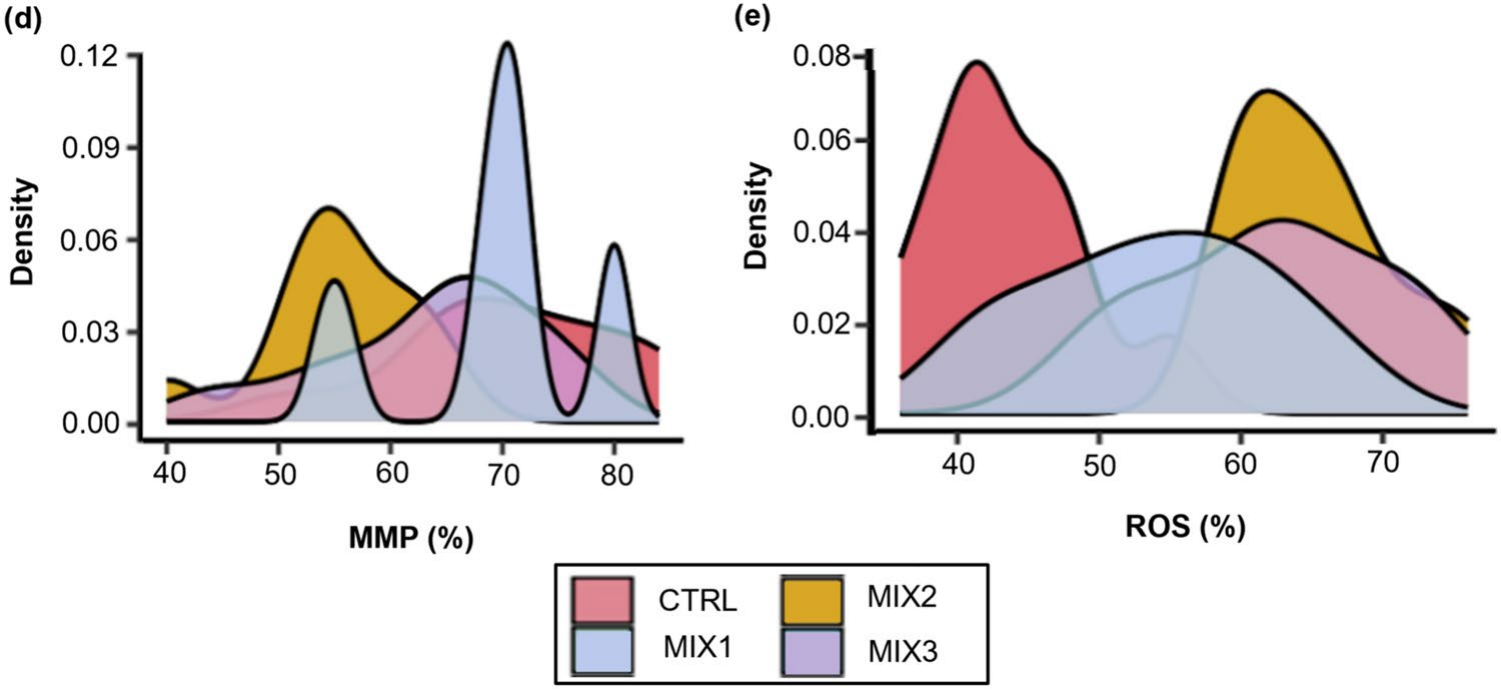
The effect of the collective mixture (containing NPX, DCF, SX, ATZ and CHL) at increasing concentrations on percentage sperm progressive motility for individual samples after 5- and 60-minutes exposure (actual averages and standard deviations are portrayed in Table S5) (**a**), sperm vitality (**b**), viability (%, normalized to control) (mean ± SD) (**c**), mitochondrial membrane potential intactness (**d**), and positive reactive oxygen species production (**e**), Note: The dot and line graph (**a**) combines dots to represent the individual sample data points and lines to connect these points, providing a visual representation of trends, patterns, or relationships over time. The box plots (**b**) provide a visual depiction of the variability present within a dataset by presenting the median, upper and lower quarltiles, as well as the minimum and maximum values and outliers. ^a^^, b, c^One-way ANOVA for parametric distributions or Kruskal-Wallis test for non-parametric distributions was used to calculate significatnt (p< 0.05) differences between mixture concentrations (**c**), The density plot (**d**, **e**) uses the Kernel density estimation creating a smoothed, continuous curve, offering an approximation of the underlying data distribution. The peak of a density plot reveals regions of where data is concentrated in a specific domain. MIX1-MIX3 are equal to increasing concentrations of the mixture (ng/L, ng/mL and μg/L), n=10. *ATZ* atrazine, *CHL* chlorpyrifos, *CTRL* HTF control, *DCF* diclofenac, *MIX* mixture, *MMP* mithochondrial membrane potential, *NPX* naproxen, *ROS* reactive oxygen species, *SD* standard deviation, *SX* sulfamethoxazole

To validate human spermatozoa as a model for toxicological assessments, motile sperm were exposed to a mixture of NPX, DCF, SX, ATZ, and CHL at environmentally relevant concentrations (MIX1: ng/L, MIX2: µg/L, MIX3: µg/mL) for 60 min. The samples were assessed for motility, kinematic parameters, vitality, viability, MMP, and ROS production (Fig. 5a-e).

After 60 min, no significant differences in motility percentages were observed between MIX1, MIX2, MIX3, and the control (Fig. 5a & Table S5). However, a dose-dependent decline in progressive motility was noted (Fig. 5a), with higher mixture concentrations causing more pronounced reductions. MIX2 (35.3 ± 9.5%) and MIX3 (28.3 ± 10.3%) significantly reduced sperm vitality compared to the control (46.7 ± 6.4%) (Fig. 5b), with MIX3 having a stronger effect. A similar trend was observed for sperm viability, where all mixtures reduced viability compared to the control group (100.0 ± 6.1%), with MIX3 (79.5 ± 12.5%) showing the greatest effect (Fig. 5c). For MMP and ROS production (Fig. 5d & 5e), MIX3 (55.2 ± 7.0%) significantly decreased MMP compared to the control (70.6 ± 10.0%), and MIX2 and MIX3 significantly increased ROS production, with MIX3 (65.2 ± 5.6%) showing the greatest increase compared to MIX1 (53.9 ± 8.4%).

## Discussion

The global chemical industry, being the second largest manufacturing sector, has registered over 70 000 new chemicals in the past decade, contributing to the rise in CECs and environmental pollution (Persson et al. 2022). Two key concerns with this soaring production capacity are the lack of toxicology studies on many CECs and underdeveloped testing techniques for detecting emerging pollutants (Ekeoma et al. 2023). Addressing these issues requires efficient, cost-effective, and sensitive screening tools to assess environmental risks.

Spermatozoa have been proposed as an effective in vitro model for assessing the impacts of antioxidants, toxins, and pollutants on physiological functions (Vollmer et al. 2019; Marcu et al. 2023; Moretti et al. 2023). As highly differentiated cells performing measurable functions for fertilization, spermatozoa offer unique advantages. However, significant variations in experimental conditions, such as donor species, sample handling, and exposure durations, complicate cross-study comparisons (Sarosiek et al. 2009; Li et al. 2010; Dietrich et al. 2012; Kollár et al. 2018). To address this, we evaluated the effects of five selected contaminants on various sperm parameters.

Our study used a highly motile human sperm subpopulation separated from seminal plasma to minimize heterogeneity and capture subtle toxicological effects (Santolaria et al. 2016; Keyser et al. 2022). This approach allowed us to track changes in sperm performance in response to environmental contaminants and identify the most reliable endpoints for chemical toxicity. We propose a new approach methodology (NAM) using human spermatozoa as an in vitro model for toxicity screening. Our findings not only confirm the high level of sperm responsiveness and preservation of motility over an hour incubation period, but also highlight the importance of identifying specific sperm functional parameters which are sensitive enough to react to environmental changes. We demonstrate that conventional semen parameters such as total motility are insufficient for capturing subtle toxicant effects, while providing detailed insights into evaluating more sophisticated endpoints such as sperm motility and kinematics, hyperactivation, vitality, viability, MMP, and ROS.

Spermatozoa responded positively to capacitating media, showing increased progressive motility, VCL, and VAP, but not total motility, which has been linked to pregnancy outcomes in intrauterine insemination (Miller et al. 2002). However, total motility is less reliable in natural conception, where spermatozoa must navigate the acidic vaginal environment and cervical barrier (Nakano et al. 2015; Sakkas et al. 2015). Our results also showed that the percentage hyperactivation was 2.5 times higher in capacitating media, suggesting improved cellular competency in this environment. By mimicking the molecular environment in vitro, we demonstrated that spermatozoa respond to external stimuli, supporting their use as a model for evaluating toxicological agents.

To evaluate spermatozoa as a toxicology model, we exposed a highly motile subpopulation to increasing contaminant concentrations. The dose-dependent effects observed across all sperm parameters over 60 min suggest distinct mechanisms of action for each chemical. All chemicals significantly reduced progressive motility but not total motility, confirming the latter's limited value in toxicological assessments. The highest concentrations of NPX, DCF, SX, and CHL had the most pronounced negative effects on progressive motility, VCL, VAP, and hyperactivation. Interestingly, AZT had the most detrimental effect at its lowest concentration, indicating a non-monotonic dose–response. The latter response is often seen in endocrine disruptors which cause a rapid signalling event that triggers the activation of nuclear transcription factors in spermatozoa leading to their dysfunctional phenotype (Vandenberg et al. 2012). These chemicals also negatively impacted vitality, viability, and MMP, disrupting traits essential for sperm survival and function. Given the link between progressive motility, VCL, VAP, MMP, and viability (Akbarinejad et al. 2020), these findings underscore the utility of such parameters in toxicology screening and early-stage evaluation of new compounds (Freires et al. 2017).

Ecosystems often face contaminant mixtures rather than single substances, with interactions potentially amplifying ecological risks through antagonistic or synergistic effects (Escher et al. 2020; Wang et al. 2024). Even chemicals below detection limits or effect thresholds can contribute to the toxicity of complex mixtures (Escher et al. 2020). Beyond establishing spermatozoa as a toxicology model, this study highlights their potential in assessing environmental risks. Exposing spermatozoa to environmentally-relevant mixtures revealed significant reductions in vitality, viability, MMP, and ROS at the highest concentrations, despite variability in progressive motility. The findings suggest spermatozoa could serve as biomarkers for contamination and inform conservation efforts, linking sperm traits to the fitness of diploid organisms (Alavioon et al. 2017; Marcu et al. 2024).

For bioanalytical tools or cellular models to effectively monitor chemical exposure, they must quantify specific, physiological, or unspecific responses at various biological levels (Busch et al. 2016; Vollmer and Stegmayr 2022). Such effects result from chemical interactions with biomolecules, altering enzyme activity, protein expression, DNA integrity, membranes, metabolism, and stress responses (Busch et al. 2016). The following section explores the potential modes of action of the five chemicals investigated in this study.

Nonsteroidal anti-inflammatory drugs (NSAIDs) like naproxen (NPX) and diclofenac (DCF) are widely used for their anti-inflammatory effects (Panchal and Sabina 2023), mediated by COX-1 and COX-2 inhibition (Boizet-Bonhoure et al. 2022; Hoxha et al. 2023). In our study, NPX and DCF exposure impaired motility, kinematics, hyperactivation, and vitality, with DCF also reducing MMP and viability. Similarly, Gallego-Ríos et al. (2021) found that NSAID exposure reduced motility and kinematics in striped catfish spermatozoa, likely due to oxidative injury and lipid peroxidation. These processes damage mitochondrial proteins, disrupt ATP production, and impair sperm motility (Dutta et al. 2021). Additionally, DCF elevates nitric oxide (NO) production, which, when excessive, can lead to oxidative stress and cell death (Banihani 2021). Interestingly, nitric oxide synthases, the enzymes responsible for producing NO, are present in human spermatozoa (O’Bryan et al. 1998; Revelli et al. 1999). While NO is critical for maintaining sperm function, it can induce cell death when excessively elevated (Francavilla et al. 2000), as in the case of DCF.

Sulfamethoxazole (SX), a widely used sulfonamide antibiotic, is effective against Gram-positive and Gram-negative bacteria (Lazzara et al. 2022). Antibiotic exposure has been linked to reduced male fertility, impacting spermatogenesis and sperm function (Mohammedi et al. 2024). In this study, SX significantly reduced sperm vitality, motility, hyperactivation, and swimming speed. Previous research showed that co-trimoxazole (SX with trimethoprim) impaired human sperm motility and viability, increasing drug sensitivity tenfold (Hargreaves et al. 1998; Salarkia et al. 2017). SX toxicity in marine organisms involved oxidative stress, immune suppression, inflammation, inhibition of acetylcholinesterase (AChE) activity, disruption of osmotic regulation and energy metabolism (Mohammedi et al. 2024). Furthermore, SX induces cytotoxicity by producing ROS and activating apoptosis and necrosis (Li et al. 2022). Spermatozoa from various animal species (e.g. fish, rabbits, bulls, and humans) exhibit AChE activity (localized to their tails) and an interaction between AChE and enolase may enhance glucose metabolism, thereby increasing ATP levels and promoting sperm motility (Mor et al. 2008). Thus, by inhibiting AChE activity and altering other enzymatic activities, SX reduces ATP production, impairing sperm motility (Nagpal & Singh 2004; Oputiri & Elias 2014).

Many pesticides act as endocrine-disrupting chemicals (EDCs), interfering with hormone function and impacting reproduction and development (Moreira et al. 2021). Atrazine (ATZ), a chlorinated triazine herbicide, is a well-known EDC used in agriculture to inhibit photosynthesis by disrupting electron transport in plants (Brown et al. 2021; Zhu et al. 2021). Despite its plant-targeted design, ATZ negatively affects non-target organisms (Poonia et al. 2022; Das et al. 2023). In this study, ATZ exposure significantly reduced sperm vitality, motility, kinematics, and hyperactivation, with stronger effects observed at lower concentrations, indicating a non-monotonic dose–response pattern (Vandenberg et al. 2012). Previous studies reported similar effects in human sperm, including reduced sperm motility and vitality, and mitochondrial ATP synthesis (Zhu et al. 2021; Das et al. 2023). In *Drosophila melanogaster*, ATZ altered the production of proteins involved in energy production, oxidative stress, and the expression of stress response genes (Brown et al. 2021). ATZ disrupts mitochondrial electron transport and induces oxidative stress, likely through inhibition of glucose-6-phosphate dehydrogenase and ATPases, as well as binding to mitochondrial ETC complexes I and II (Brown et al. 2021; Abarikwu et al. 2023; Das et al. 2023).

Chlorpyrifos (CHL), an organophosphate pesticide, induces neurotoxicity by inhibiting AChE, leading to acetylcholine accumulation and nervous system collapse in insects (Alaa-Eldin et al. 2017; Bosu et al. 2024). However, it also causes hepatotoxicity, nephrotoxicity, and reproductive toxicity in non-target organisms (Zhang et al. 2020; Bhende et al. 2022) and other putative mechanisms may include oxidative stress (Alaa-Eldin et al. 2017). In this study, CHL exposure significantly reduced human sperm motility, kinematics, hyperactivation, and vitality across all concentrations, indicating its detrimental effect on sperm function. CHL may impair male fertility by affecting sexual organ weight and sperm parameters, possibly through endocrine-disrupting properties (Abdel-Razik et al. 2021). In vivo studies in rats showed reduced follicle-stimulating hormone (FSH), luteinizing hormone (LH) and testosterone levels, along with decreased sperm motility, viability, and count, all linked to mitochondrial dysfunction, altered fructose synthesis and structural damage (Alaa-Eldin et al. 2017). Similarly, CHL exposure in fish spermatozoa decreased motility and increased oxidative stress (Kutluyer et al. 2019).

In summary, this study has proposed, established and validated an in vitro toxicology screening model using human spermatozoa based on three principles: 1) selecting a motile sperm subpopulation, 2) employing standardized and reproducible protocols to assess sperm parameters, and 3) incorporating a comprehensive panel of parameters reflecting overall sperm function. Two sperm functional aspects or assessments that have not been used in previous studies reporting on the effect of contaminants on sperm function are hyperactivation and viability (via cytotoxicity assay), both of which were particularly sensitive to toxicant effects (Hardneck et al. 2018, 2021). Spermatozoa’s sensitivity to the chemicals’ modes of action likely arises from shared enzymes and pathways with other cells, making them vulnerable to oxidative stress, lipid peroxidation, and membrane damage. In fact, mammalian gametes express unique sperm- or gamete-specific isoforms of enzymes regulating the energy pathways (Miki et al. 2004; Odet et al. 2008), thus underscoring their potential as robust bioanalytical tools.

## Conclusion

Spermatozoa, as highly specialized cells, can serve as a versatile in vitro tool for toxicological assessment. The next step is to integrate these cells into a universal toxicology model capable of screening and ranking the toxicity of various substances in a single setup. Our findings highlight the importance of selecting advanced investigative parameters, optimizing experimental conditions, and standardizing criteria for assessing physiological impacts. By focusing on advanced sperm functional parameters and considering both the contaminant concentration and exposure duration, it should be possible to standardize the assessment criteria for the physiological impacts of environmental factors. In the future, this model could be extended to evaluate the effectiveness of intervention measures post-exposure, particularly to predict whether the detrimental effects observed are permanent or reversible. Furthermore, understanding the molecular features involved offers the possibility to develop additional genetic screening tools with wider applications for toxicology and human health and disease. This approach holds promise for advancing both environmental and biomedical research.

## Supplementary Information

Below is the link to the electronic supplementary material.

Supplementary file1 (PDF 428 KB)

## Data Availability

The authors confirm that the data supporting the findings of this study are available in the article and its supplementary material.

## References

[CR1] Abarikwu SO, Ezim OE, Ikeji CN, Farombi EO (2023) Atrazine: cytotoxicity, oxidative stress, apoptosis, testicular effects and chemo-preventive Interventions. Front Toxicol 5:1246708. 10.3389/ftox.2023.1246708 37876981 10.3389/ftox.2023.1246708 PMC10590919

[CR2] Abdel-Razik RK, Mosallam EM, Hamed NA, Badawy ME, Abo-El-Saad MM (2021) Testicular deficiency associated with exposure to cypermethrin, imidacloprid, and chlorpyrifos in adult rats. Environ Toxicol Pharmacol 87:103724. 10.1016/j.etap.2021.103724 34416397 10.1016/j.etap.2021.103724

[CR3] Aitken RJ (1990) Development of *in vitro* tests of human sperm function: a diagnostic tool and model system for toxicological analyses. In Vitro Toxicol 4(4–5):560–569. 10.1016/0887-2333(90)90116-B 10.1016/0887-2333(90)90116-b 20702230

[CR4] Akbarinejad V, Fathi R, Shahverdi A, Esmaeili V, Rezagholizadeh A, Ghaleno LR (2020) The relationship of mitochondrial membrane potential, reactive oxygen species, adenosine triphosphate content, sperm plasma membrane integrity, and kinematic properties in warmblood stallions. J Equine Vet Sci 94:103267. 10.1016/j.jevs.2020.103267 33077084 10.1016/j.jevs.2020.103267

[CR5] Alaa-Eldin EA, El-Shafei DA, Abouhashem NS (2017) Individual and combined effect of chlorpyrifos and cypermethrin on reproductive system of adult male albino rats. Environ Sci Pollut Res 24:1532–1543. 10.1007/s11356-016-7912-6 10.1007/s11356-016-7912-6 27785720

[CR6] Alavioon G, Hotzy C, Nakhro K, Rudolf S, Scofield DG, Zajitschek S, Maklakov AA, Immler S (2017) Haploid selection within a single ejaculate increases offspring fitness. Proc Natl Acad Sci USA 114(30):8053–8058. 10.1073/pnas.1705601114 28698378 10.1073/pnas.1705601114 PMC5544320

[CR7] Banihani SA (2021) Effect of diclofenac on semen quality: a review. Andrologia 53(5):e14021. 10.1111/and.14021 33650710 10.1111/and.14021

[CR8] Bhende RS, Jhariya U, Srivastava S, Bombaywala S, Das S, Dafale NA (2022) Environmental distribution, metabolic fate, and degradation mechanism of chlorpyrifos: recent and future perspectives. Appl Biochem Biotechnol 194:2301–2335. 10.1007/s12010-021-03713-7 35013924 10.1007/s12010-021-03713-7

[CR9] Björndahl et al (2022) Standards in semen examination: publishing reproducible and reliable data based on high-quality methodology. Hum Reprod 37(11):2497–2502. 10.1093/humrep/deac189 36112046 10.1093/humrep/deac189 PMC9627864

[CR10] Boizet-Bonhoure B, Déjardin S, Rossitto M, Poulat F, Philibert P (2022) Using experimental models to decipher the effects of acetaminophen and NSAIDs on reproductive development and health. Front Toxicol 4:835360. 10.3389/ftox.2022.835360 35295217 10.3389/ftox.2022.835360 PMC8915900

[CR11] Boshoff NH, Lambrechts H, Maree L, Cloete SWP, Van der Horst G (2018) A novel flush technique to simulate natural dispersal of spermatozoa in the female reproductive tract and expedite motility assessment of fresh ejaculated Merino (Ovis aries) sperm. S Afr J Anim Sci 48(3):469–476. 10.4314/sajas.v48i3.7

[CR12] Bosu S, Rajamohan N, Al Salti S, Rajasimman M, Das P (2024) Biodegradation of chlorpyrifos pollution from contaminated environment-A review on operating variables and mechanism. Environ Res 248:118212. 10.1016/j.envres.2024.118212 38272293 10.1016/j.envres.2024.118212

[CR13] Brown JB, Langley SA, Snijders AM, Wan KH, Morris SNS, Booth BW, Fisher WW, Hammonds AS, Park S, Weiszmann R, Yu C (2021) An integrated host-microbiome response to atrazine exposure mediates toxicity in Drosophila. Commun Biol 4(1):1324 34819611 10.1038/s42003-021-02847-y PMC8613235

[CR14] Busch W, Schmidt S, Kühne R, Schulze T, Krauss M, Altenburger R (2016) Micropollutants in European rivers: a mode of action survey to support the development of effect-based tools for water monitoring. Environ Toxicol Chem 35(8):1887–1899. 10.1002/etc.3460 27299692 10.1002/etc.3460

[CR15] Castellini C, Di Giammarco N, D’Andrea S, Parisi A, Totaro M, Francavilla S, Francavilla F, Barbonetti A (2021) Effects of bisphenol S and bisphenol F on human spermatozoa: An in vitro study. Reprod Toxicol 103:58–63. 10.1016/j.reprotox.2021.05.011 34089804 10.1016/j.reprotox.2021.05.011

[CR16] Chiu YH, Afeiche MC, Gaskins AJ, Williams PL, Petrozza JC, Tanrikut C, Hauser R, Chavarro JE (2022) Association between pesticide residue intake from consumption of fruits and vegetables and pregnancy outcomes among women undergoing infertility treatment with assisted reproductive technology. JAMA Intern Med 178(1):17–26. 10.1001/jamainternmed.2017.5038 10.1001/jamainternmed.2017.5038 PMC5814112 29084307

[CR17] Das S, Sakr H, Al-Huseini I, Jetti R, Al-Qasmi S, Sugavasi R, Sirasanagandla SR (2023) Atrazine toxicity: the possible role of natural products for effective treatment. Plants 12(12):2278. 10.3390/plants12122278 37375903 10.3390/plants12122278 PMC10301673

[CR18] Diamond JM, Latimer HA II, Munkittrick KR, Thornton KW, Bartell SM, Kidd KA (2011) Prioritizing contaminants of emerging concern for ecological screening assessments. Environ Toxicol Chem 30:2385–2394 22002713 10.1002/etc.667

[CR19] Dietrich GJ, Ciereszko A, Kowalski RK, Rzemieniecki A, Bogdan E, Demianowicz W, Dietrich M, Kujawa R, Glogowski J (2012) Motility and fertilizing capacity of frozen/thawed sperm of S iberian sturgeon after a short-time exposure of fresh semen to mercury and cadmium. J Appl Ichthyol 28(6):973–977. 10.1111/jai.12062

[CR20] Dutta S, Sengupta P, Slama P, Roychoudhury S (2021) Oxidative stress, testicular inflammatory pathways, and male reproduction. Int J Mol Sci 22(18):10043. 10.3390/ijms221810043 34576205 10.3390/ijms221810043 PMC8471715

[CR21] Ekeoma BC, Ekeoma LN, Yusuf M, Haruna A, Ikeogu CK, Merican ZMA, Kamyab H, Pham CQ, Vo DN, Chelliapan S (2023) Recent advances in the biocatalytic mitigation of emerging pollutants: a comprehensive review. J Biotech 369:14–34. 10.1016/j.jbiotec.2023.05.003 10.1016/j.jbiotec.2023.05.003 37172936

[CR23] Escher BI, Stapleton HM, Schymanski EL (2020) Tracking complex mixtures of chemicals in our changing environment. Science 367(6476):388–392. 10.1126/science.aay6636 31974244 10.1126/science.aay6636 PMC7153918

[CR24] Escher BI, Altenburger R, Blüher M, Colbourne JK, Ebinghaus R, Fantke P, Hein M, Köck W, Kümmerer K, Leipold S, Li X (2023) Modernizing persistence–bioaccumulation–toxicity (PBT) assessment with high throughput animal-free methods. Arch Toxicol 97(5):1267–1283. 10.1007/s00204-023-03485-5 36952002 10.1007/s00204-023-03485-5 PMC10110678

[CR25] Francavilla F, Santucci R, Macerola B, Ruvolo G, Romano R (2000) Nitric oxide synthase inhibition in human sperm affects sperm-oocyte fusion but not zona pellucida binding. Biol Reprod 63(2):425–429. 10.1095/biolreprod63.2.425 10906046 10.1095/biolreprod63.2.425

[CR26] Freires IA, Sardi JDCO, de Castro RD, Rosalen PL (2017) Alternative animal and non-animal models for drug discovery and development: bonus or burden? Pharm Res 34:681–686. 10.1007/s11095-016-2069-z 27858217 10.1007/s11095-016-2069-z

[CR27] Freires IA, Morelo DFC, Soares LFF, Costa IS, de Araújo LP, Breseghello I, Abdalla HB, Lazarini JG, Rosalen PL, Pigossi SC, Franchin M (2023) Correction to: Progress and promise of alternative animal and non-animal methods in biomedical research. Arch Toxicol 97(11):3021. 10.1007/s00204-023-03595-0 37665364 10.1007/s00204-023-03595-0

[CR28] Gallego-Ríos SE, Atencio-García VJ, Peñuela GA (2021) Effect of ibuprofen in vivo and in vitro on the sperm quality of the striped catfish Pseudoplatystoma magdaleniatum. Environ Sci Pollut Res 28:36133–36141. 10.1007/s11356-021-13245-6 10.1007/s11356-021-13245-6 33683592

[CR29] Ghersevich S, Massa E, Zumoffen C (2015) Oviductal secretion and gamete interaction. Reproduction 149(1):R1–R14. 10.1530/REP-14-0145 25190504 10.1530/REP-14-0145

[CR30] Gruber FS, Johnston ZC, Barratt CLR, Andrews PD (2020) A phenotypic screening platform utilising human spermatozoa identifies compounds with contraceptive activity. Elife 9:351739 10.7554/eLife.51739 PMC7046468 31987071

[CR31] Hardneck F, Israel G, Pool E, Maree L (2018) Quantitative assessment of heavy metal effects on sperm function using computer-aided sperm analysis and cytotoxicity assays. Andrologia 50(10):e13141. 10.1111/and.13141 30225848 10.1111/and.13141

[CR32] Hardneck F, de Villiers C, Maree L (2021) Effect of copper sulphate and cadmium chloride on non-human primate sperm function in vitro. Int J Environ Res Public Health 18:6200. 10.3990/ijerph18126200 34201151 10.3390/ijerph18126200 PMC8228149

[CR33] Hargreaves CA, Rogers S, Hills F, Rahman F, Howell RJ, Homa ST (1998) Effects of co-trimoxazole, erythromycin, amoxycillin, tetracycline and chloroquine on sperm function in vitro. Hum Reprod 13(7):1878–1886. 10.1093/humrep/13.7.1878 9740442 10.1093/humrep/13.7.1878

[CR34] Holt EA, Miller SW (2011) Bioindicators: Using Organisms to Measure Environmental Impacts. Nature Education Knowledge 2(2):8

[CR35] Hoxha M, Barbonetti A, Zappacosta B (2023) Arachidonic acid pathways and male fertility: a systematic review. Int J Mol Sci 24(9):8207. 10.3390/ijms24098207 37175913 10.3390/ijms24098207 PMC10178949

[CR36] Jain AK, Singh D, Dubey K, Maurya R, Mittal S, Pandey AK (2018) Models and methods for in vitro toxicity. In Vitro Toxicol. 10.1016/B978-0-12-804667-8.00003-1

[CR37] Jeng HA (2014) Exposure to endocrine disrupting chemicals and male reproductive health. Front Public Health 2:55. 10.3389/fpubh.2014.00055 24926476 10.3389/fpubh.2014.00055 PMC4046332

[CR38] Kassotis C, Stapleton HM (2019) Endocrine-Mediated Mechanisms of Metabolic Disruption and New Approaches to Examine the Public Health Threat. Front Endocrinol 10:39. 10.3389/fendo.2019.00039 10.3389/fendo.2019.00039 PMC6374316 30792693

[CR39] Keyser S, Van Der Horst G, Maree L (2022) New approaches to define the functional competency of human sperm subpopulations and its relationship to semen quality. Int J Fertil Steril 16(3):140 36029048 10.22074/IJFS.2021.531517.1132 PMC9396000

[CR40] Kollár T, Kása E, Csorbai B, Urbányi B, Csenki-Bakos Z, Horváth Á (2018) In vitro toxicology test system based on common carp (Cyprinus carpio) sperm analysis. Fish Physiol Biochem 44:1577–1589. 10.1007/s10695-018-0541-x 30043206 10.1007/s10695-018-0541-x

[CR41] Kortenkamp A, Faust M (2018) Regulate to reduce chemical mixture e risk. Science 361(6399):224–226. 10.1126/science.aat9219 30026211 10.1126/science.aat9219

[CR42] Kosnik MB, Hauschild MZ, Fantke P (2022) Toward assessing absolute environmental sustainability of chemical pollution. Environ Sci Technol 56(8):4776–4787. 10.1021/acs.est.1c06098 35349278 10.1021/acs.est.1c06098 PMC9022439

[CR43] Kotwicka M, Skibinska I, Piworun N, Jendraszak M, Chmielewska M, Jedrzejczak P (2016) Bisphenol A modifies human spermatozoa motility in vitro. J Med Sci 85(1):39–45

[CR44] Kutluyer F, Kocabaş M, Erişir M, Benzer F (2019) Effect of the organophosphate insecticide chlorpyrifos exposure on oxidative stress and quality of Salmo coruhensis spermatozoa. Toxin Rev 38(1):71–76. 10.1080/15569543.2017.1394325

[CR45] Lazzara V, Mauro M, Celi M, Cammilleri G, Vizzini A, Luparello C, Bellini P, Ferrantelli V, Vazzana M (2022) Effects of Sulfamethoxazole on Fertilization and Embryo Development in the Arbacia lixula Sea Urchin. Animals 12(18):2483. 10.3390/ani12182483 36139342 10.3390/ani12182483 PMC9495157

[CR46] Li ZH, Li P, Dzyuba B, Randak T (2010) Influence of environmental related concentrations of heavy metals on motility parameters and antioxidant responses in sturgeon sperm. Chem -Biol Interact 188(3):473–477. 10.1016/j.cbi.2010.09.005 20836996 10.1016/j.cbi.2010.09.005

[CR47] Li B, Wang Y, Zhao H, Yin K, Liu Y, Wang D, Zong H, Xing M (2022) Oxidative stress is involved in the activation of NF-κB signal pathway and immune inflammatory response in grass carp gill induced by cypermethrin and/or sulfamethoxazole. Environ Sci Pollut Res. 10.1007/s11356-021-17197-9 10.1007/s11356-021-17197-9 34718981

[CR48] López-Botella A, Velasco I, Acién M, Sáez-Espinosa P, Todolí-Torró JL, Sánchez-Romero R, Gómez-Torres MJ (2021) Impact of heavy metals on human male fertility-an overview. Antioxidants (Basel) 9:1473. 10.3390/antiox10091473 10.3390/antiox10091473 PMC8468047 34573104

[CR49] Madorran E, Stožer A, Bevc S, Maver U (2020) *In vitro* toxicity model: Upgrades to bridge the gap between preclinical and clinical research. Biomol Biomed 20(2):157–168 10.17305/bjbms.2019.4378 PMC7202182 31621554

[CR50] Marcu D, Keyser S, Petrik L, Fuhrimann S, Maree L (2023) Contaminants of Emerging Concern (CECs) and Male Reproductive Health: Challenging the Future with a Double-Edged Sword. Toxics 11(4):330. 10.3390/toxics11040330 37112557 10.3390/toxics11040330 PMC10141735

[CR51] Marcu D, Cohen-Krais J, Godden A, Alavioon G, Martins C, Almstrup K, Saalbach G, Immler S (2024) Within-ejaculate haploid selection reduces disease biomarkers in human sperm. bioRxiv. 10.1101/2024.10.08.617222

[CR52] Microptic S.L. (2022) Vitality slides SCA Protocol. Available from: https://www.micropticsl.com/documents-support/protocols/. Accessed 5 May 2024

[CR53] Miki K, Qu W, Goulding EH, Willis WD, Bunch DO, Strader LF, Perreault SD, Eddy EM, O’Brien DA (2004) Glyceraldehyde 3-phosphate dehydrogenase-S, a sperm-specific glycolytic enzyme, is required for sperm motility and male fertility. Proc Natl Acad Sci 101(47):16501–16506. 10.1073/pnas.0407708101 15546993 10.1073/pnas.0407708101 PMC534542

[CR54] Miller DC, Hollenbeck BK, Smith GD, Randolph JF, Christman GM, Smith YR, Lebovic DI, Ohl DA (2002) Processed total motile sperm count correlates with pregnancy outcome after intrauterine insemination. Urology 60(3):497–501. 10.1016/S0090-4295(02)01773-9 12350496 10.1016/s0090-4295(02)01773-9

[CR55] Mohammedi L, Messaï A, Ouamane H, Bencharif S, Touazi L, Iguer-Ouada M (2024) *In vivo* effect of ampicillin, enrofloxacin, colistin, and sulfonamides on sperm parameters in breeding roosters. J Microbiol Biotechnol Food Sci 13(5):e9607

[CR56] Mor I, Sklan EH, Podoly E, Pick M, Kirschner M, Yogev L, Bar-Sheshet Itach S, Schreiber L, Geyer B, Mor T, Grisaru D (2008) Acetylcholinesterase-R increases germ cell apoptosis but enhances sperm motility. J Cell Mol Med 12(2):479–495. 10.1111/j.1582-4934.2008.00231.x 18194455 10.1111/j.1582-4934.2008.00231.x PMC3822537

[CR57] Moreira S, Pereira SC, Seco-Rovira V, Oliveira PF, Alves MG, Pereira MDL (2021) Pesticides and male fertility: A dangerous crosstalk. Metabolites 11(12):799. 10.3390/metabo11120799 34940557 10.3390/metabo11120799 PMC8707831

[CR58] Moretti E, Signorini C, Corsaro R, Giamalidi M, Collodel G (2023) Human Sperm as an *In Vitro* Model to Assess the Efficacy of Antioxidant Supplements during sperm Handling: A Narrative Review. Antioxidants 12:1098. 10.3390/antiox12051098 37237965 10.3390/antiox12051098 PMC10215929

[CR59] Mortimer D (1994) Practical Laboratory Andrology. Oxford University Press on Demand, New York

[CR60] Mortimer D (2018) The functional anatomy of the human spermatozoon: relating ultrastructure and function. Mol Hum Reprod 24(12):567–592. 10.1093/molehr/gay040 30215807 10.1093/molehr/gay040

[CR61] Mortimer D, Mortimer ST (2013) Computer-Aided Sperm Analysis (CASA) of Sperm Motility and Hyperactivation. In: Carrell D, Aston K (eds) Spermatogenesis, Methods in Molecular Biology, vol 927. Humana Press. Totowa, NJ, pp 77–87 10.1007/978-1-62703-038-0_8 22992905

[CR62] Nagpal P, Singh RV (2004) Toxicological effects, biological aspects and spectral characterization of organoboron (III) complexes of sulfonamide-imines. Appl Organomet Chem 18(5):221–226. 10.1002/aoc.610

[CR63] Naidu R, Biswas B, Willett IR, Cribb J, Singh BK, Nathanail CP, Coulon F, Semple KT, Jones KC, Barclay A, Aitken RJ (2021) Chemical pollution: a growing peril and potential catastrophic risk to humanity. Environ Int 156:106616. 10.1016/j.envint.2021.106616 33989840 10.1016/j.envint.2021.106616

[CR64] Nakano FY, Leão RDBF, Esteves SC (2015) Insights into the role of cervical mucus and vaginal pH in unexplained infertility. MedicalExpress 2(2):M150207. 10.5935/MedicalExpress.2015.02.07

[CR65] Neale PA, Braun G, Brack W, Carmona E, Gunold R, König M, Krauss M, Liebmann L, Liess M, Link M, Schäfer RB, Schlichting R, Schreiner VC, Schulze T, Vormeier P, Weisner O, Escher BI (2020) Assessing the Mixture Effects in *In Vitro* Bioassays of Chemicals Occurring in Small Agricultural Streams during Rain Events. Environ Sci Technol 54(13):8280–8290. 10.1021/acs.est.0c02235 32501680 10.1021/acs.est.0c02235

[CR66] U.S. NAS (2017) Using 21st century science to improve risk-related evaluations. The National Academies Press, Washington, DC. 10.17226/24635. 28267305

[CR67] O’Bryan MK, Zini A, Cheng CY, Schlegel PN (1998) Human sperm endothelial nitric oxide synthase expression: correlation with sperm motility. Fertil Steril 70(6):1143–1147. 10.1016/S0015-0282(98)00382-3 9848308 10.1016/s0015-0282(98)00382-3

[CR68] Odet F, Duan C, Willis W, Goulding E, Kung A, Eddy M, Goldberg E (2008) Lactate dehydrogenase-C-4 (LDH-C-4) is essential for sperm function. Biol Reprod 78(1):187 10.1095/biolreprod.108.068353 PMC2574787 18367675

[CR69] Ojemaye CY, Petrik L (2022) Pharmaceuticals and personal care products in the marine environment around False Bay, Cape Town, South Africa: Occurrence and risk—Assessment study. Environ Toxicol Chem 41:614–634. 10.1002/etc.5053 33783837 10.1002/etc.5053

[CR70] Ojemaye CY, Onwordi CT, Pampanin DM, Sydnes MO, Petrik L (2020) Presence and risk assessment of herbicides in the marine environment of Camps Bay (Cape Town, South Africa). Sci Total Environ 738:140346. 10.1016/j.scitotenv.2020.140346 32806370 10.1016/j.scitotenv.2020.140346

[CR71] Ojemaye CY, Onwordi CT, Pampanin DM, Sydnes MO, Petrik L (2021) Herbicides in Camps Bay (Cape Town, South Africa), supplemented. Sci Total Environ 778:146057. 10.1016/j.scitotenv.2021.146057 33714098 10.1016/j.scitotenv.2021.146057

[CR72] Oputiri D, Elias A (2014) Impact of co-administered lopinavir/ritonavir and sulfamethoxazole/trimethoprim on reproductive indices of male albino rats. Am J Pharmacol Sci 2(5):93–99

[CR73] Panchal NK, Sabina EP (2023) Non-steroidal anti-inflammatory drugs (NSAIDs): A current insight into its molecular mechanism eliciting organ toxicities. Food Chem Toxicol 172:113598. 10.1016/j.fct.2022.113598 36608735 10.1016/j.fct.2022.113598

[CR74] Persson L, Carney Almroth BM, Collins CD, Cornell S, De Wit CA, Diamond ML, Fantke P, Hassellöv M, MacLeod M, Ryberg MW, Søgaard Jørgensen P (2022) Outside the safe operating space of the planetary boundary for novel entities. Environ Sci Technol 56(3):1510–1521. 10.1021/acs.est.1c04158 35038861 10.1021/acs.est.1c04158 PMC8811958

[CR75] Petrik L, Green L, Abegunde AP, Zackon M, Sanusi CY, Barnes J (2017) Desalination and seawater quality at Green Point, Cape Town: A study on the effects of marine sewage outfalls. S Afr J Sci 113:1–10

[CR76] Poonia K, Hasija V, Singh P, Khan AAP, Thakur S, Thakur VK, Mukherjee S, Ahamad T, Alshehri SM, Raizada P (2022) Photocatalytic degradation aspects of atrazine in water: Enhancement strategies and mechanistic insights. J Clean Prod 367:133087. 10.1016/j.jclepro.2022.133087

[CR77] Revelli A, Soldati G, Costamagna C, Pellerey O, Aldieri E, Massobrio M, Bosia A, Ghigo D (1999) Follicular fluid proteins stimulate nitric oxide (NO) synthesis in human sperm: a possible role for NO in acrosomal reaction. J Cell Physiol 178(1):85–92 9886494 10.1002/(SICI)1097-4652(199901)178:1<85::AID-JCP11>3.0.CO;2-Y

[CR78] Sakkas D, Ramalingam M, Garrido N, Barratt CLR (2015) Sperm selection in natural conception: what can we learn from Mother Nature to improve assisted reproduction outcomes? Hum Reprod Update 21(6):711–726. 10.1093/humupd/dmv042 26386468 10.1093/humupd/dmv042 PMC4594619

[CR79] Salarkia E, Sepehri G, Torabzadeh P, Abshenas J, Saberi A (2017) Effects of administration of co-trimoxazole and folic acid on sperm quality and histological changes of testes in male rats. Int J Reprod Biomed 15(10):625–634 29387828 PMC5767643

[CR80] Santolaria P, Soler C, Recreo P, Carretero T, Bono A, Berné JM, Yániz JL (2016) Morphometric and kinematic sperm subpopulations in split ejaculates of normozoospermic men. Asian J Androl 18(6):831–834. 10.4103/1008-682X.186874 27624985 10.4103/1008-682X.186874 PMC5109871

[CR81] Sarosiek B, Pietrusewicz M, Radziwoniuk J, Glogowski J (2009) The effect of copper, zinc, mercury and cadmium on some sperm enzyme activities in the common carp (Cyprinus carpio L.). Reprod Biol 9(3):295–301 19997481 10.1016/s1642-431x(12)60033-3

[CR82] Sauvé S, Desrosiers M (2014) A review of what is an emerging contaminant. Chem Cent J 8:1–7. 10.1186/1752-153X-8-15 24572188 10.1186/1752-153X-8-15 PMC3938815

[CR83] Shaliutina O, Materiienko A, Shaliutina-Kolešová A, Gazo I (2021) Using fish spermatozoa in in vitro toxicity tests: a potential toxicology tool. Aquaculture 539:736647. 10.1016/j.aquaculture.2021.736647

[CR84] Sysoeva AP, Nepsha OS, Makarova NP, Silachev DN, Lobanova NN, Timofeeva AV, Shevtsova YA, Bragina EE, Kalinina EA (2022) Influence of extracellular vesicles from the follicular fluid of young women and women of advanced maternal age with different miRNA profiles on sperm functional properties. Bulletin Exp Biol Med 173(4):560–568. 10.1007/s10517-022-05589-x 10.1007/s10517-022-05589-x 36094592

[CR22] U.S. EPA (2021) New Approach Methods Work Plan (v2). U.S. Environmental Protection Agency, Washington, DC. EPA/600/X-21/209.

[CR85] Vallaeys T, Klink SP, Fleouter E, Le Moing B, Lignot JH, Smith AJ (2017) Bioindicators of marine contaminations at the frontier of environmental monitoring and environmental genomics. Adv Biotech Micro 4(1):555629

[CR86] Vandenberg LN, Colborn T, Hayes TB, Heindel JJ, Jacobs DR Jr, Lee DH, Shioda T, Soto AM, vom Saal FS, Welshons WV, Zoeller RT (2012) Hormones and endocrine-disrupting chemicals: low-dose effects and nonmonotonic dose responses. Endocr Rev 33(3):378–455. 10.1210/er.2011-1050 22419778 10.1210/er.2011-1050 PMC3365860

[CR87] Vollmer T, Stegmayr B (2022) Establishing Cell Models to Understand Cellular Toxicity: Lessons Learned from an Unconventional Cell Type. Toxins 14(1):54. 10.3390/toxins14010054 35051031 10.3390/toxins14010054 PMC8779380

[CR88] Vollmer T, Ljungberg B, Jankowski V, Jankowski J, Glorieux G, Stegmayr BG (2019) An *in-vitro* assay using human spermatozoa to detect toxicity of biologically active substances. Sci Rep 9(1):14525. 10.1038/s41598-019-50929-z 31601841 10.1038/s41598-019-50929-z PMC6787250

[CR89] Wang F, Xiang L, Leung KSY et al (2024) Emerging contaminants: a One Health perspective. The Innovation 5(4):100612. 10.1016/j.xinn.2024.100612 38756954 10.1016/j.xinn.2024.100612 PMC11096751

[CR90] WHO (2021) WHO Laboratory Manual for the Examination and Processing of Human Semen, 6th edn. World Health Organization, Geneva

[CR91] World Medical Association (2013) World Medical Association Declaration of Helsinki: ethical principles for medical research involving human subjects. JAMA 310(20):2191–2194. 10.1001/jama.2013.281053 24141714 10.1001/jama.2013.281053

[CR92] Zhang X, Cui W, Wang KE, Chen R, Chen M, Lan K, Wei Y, Pan C, Lan X (2020) Chlorpyrifos inhibits sperm maturation and induces a decrease in mouse male fertility. Environ Res 188:109785 32798940 10.1016/j.envres.2020.109785

[CR93] Zhu S, Zhang T, Wang Y, Zhou X, Wang S, Wang Z (2021) Meta-analysis and experimental validation identified atrazine as a toxicant in the male reproductive system. Environ Sci Pollut Res 28:37482–37497. 10.1007/s11356-021-13396-6 10.1007/s11356-021-13396-6 33715114

