# Supplementary material for: Human sperm as an in vitro toxicity model: a versatile tool for assessing the risk of environmental contaminants
Source: Arch Toxicol. 2025 May 3;99(7):2937–52. doi: 10.1007/s00204-025-04035-x (PMC12198078; doi:10.1007/s00204-025-04035-x)
Supplement: Supplementary file 1 — Supplementary file1 (PDF 428 KB) [file 204_2025_4035_MOESM1_ESM.pdf]

Supplementary Material:

Archives of Toxicology

Human sperm as an *in vitro* toxicity model: a versatile tool for assessing the risk of environmental contaminants

Shannen Keyser[1,3*], Daniel Marcu[2*], Morgan T.D. Davidse[3], Monique Bennett[3], Leslie Petrik[4], Liana Maree[3]

[1] School of Nursing, Faculty of Community and Health Sciences, University of the Western Cape, Private Bag X17, Bellville 7535, South Africa

[2] School of Biological Sciences, University of East Anglia, NR4 7TJ, United Kingdom

[3] Comparative Spermatology Laboratory, Department of Medical Bioscience, University of the Western Cape, Private Bag X17, Bellville 7535, South Africa

[4] Environmental and Nano Sciences Group, Department of Chemistry, University of the Western Cape, Private Bag X17, Bellville 7535, South Africa

***Authors contributed equally to the work**

Corresponding author: Liana Maree; lmaree@uwc.ac.za; https://orcid.org/0000-0003-4147-4221

Table S1: Basic semen analysis of 58 donor semen samples (n=58) (mean ± SD).

	Mean ± SD
**Volume (mL)**	3.3 ± 1.3
**pH**	7.5 ± 0.6
**Total Motility (%)**	51.1 ± 15.5
**Progressive Motility (%)**	25.9 ± 12.2
**CMP (mil/ejaculate)**	13.6 ± 14.5
**Concentration (mil/mL)**	34.7 ± 23.9

CMP , cervical mucus penetration test; *SD* , standard deviation

Table S2: Motility parameters of human spermatozoa after 5-, 30- and 60-minutes exposure to different concentrations of naproxen, diclofenac, sulfamethoxazole, atrazine and chlorpyrifos (n=10) (mean ± SD).

	Treatment						ANOVA
	Control	CAP	T1	T2	T3	T4	
**Total Mot (%)**							
**5 min**							
**NPX**	86.8 ± 10.4	91.4 ± 8.9	77.0 ± 15.5	76.0 ± 19.1	80.2 ± 14.2	75.0 ± 13.3	0.061
**DCF**	86.8 ± 10.4^{ab}	91.4 ± 8.9^{b}	76.0 ± 17.4^{ad}	69.7 ± 20.1^{cd}	63.9 ± 21.6^{cd}	56.9 ± 20.9^{c}	**< 0.001**
**SX**	86.8 ± 10.4^{a}	91.4 ± 8.9^{a}	86.3 ± 6.3^{a}	85.3 ± 14.3^{a}	73.1 ± 11.1^{b}	62.9 ± 26.0^{b}	**0.002**
**ATZ**	86.8 ± 10.4^{a}	91.4 ± 8.9^{a}	51.4 ± 18.4^{b}	81.6 ± 14.9^{a}	81.0 ± 10.0^{a}	76.7 ± 9.8^{a}	**< 0.001**
**CHL**	86.8 ± 10.4^{a}	91.4 ± 8.9^{a}	26.3 ± 14.9^{b}	35.5 ± 15.0^{b}	70.2 ± 12.7^{c}	30.7 ± 11.8^{b}	**< 0.001**
**30 min**							
**NPX**	85.9 ± 8.1^{ab}	90.7 ± 7.2^{a}	76.9 ± 17.2^{ab}	83.4 ± 7.0^{ab}	77.8 ± 15.5^{ab}	74.8 ± 10.4^{b}	**0.027**
**DCF**	85.9 ± 8.1^{a}	90.7 ± 7.2^{a}	74.9 ± 15.0^{b}	67.2 ± 17.8^{bc}	65.2 ± 20.0^{bc}	54.9 ± 19.1^{c}	**< 0.001**
**SX**	85.9 ± 8.1^{ac}	90.7 ± 7.2^{a}	80.4 ± 11.0^{c}	78.1 ± 16.9^{c}	63.0 ± 11.6^{d}	45.5 ± 18.1^{d}	**< 0.001**
**ATZ**	85.9 ± 8.1^{ab}	90.7 ± 7.2^{b}	40.2 ± 14.3^{c}	79.5 ± 11.4^{ab}	83.3 ± 10.1^{ab}	74.2 ± 10.5^{a}	**< 0.001**
**CHL**	85.9 ± 8.1^{a}	90.7 ± 7.2^{a}	30.3 ± 16.1^{b}	32.6 ± 10.0^{b}	58.3 ± 15.3^{c}	37.2 ± 15.9^{b}	**< 0.001**
**60 min**							
**NPX**	82.6 ± 12.0^{ab}	88.1 ± 9.2^{a}	68.8 ± 21.3^{b}	70.2 ± 17.4^{b}	76.5 ± 11.3^{ab}	75.9 ± 9.4^{ab}	**0.030**
**DCF**	82.6 ± 12.0^{a}	88.1 ± 9.2^{a}	72.4 ± 12.2^{b}	68.1 ± 17.7^{bc}	51.4 ± 25.1^{c}	57.9 ± 18.1^{c}	**< 0.001**
**SX**	82.6 ± 12.0^{a}	88.1 ± 9.2^{a}	80.0 ± 7.3^{a}	75.1 ± 17.8^{a}	61.0 ± 13.3^{b}	43.3 ± 15.1^{c}	**< 0.001**
**ATZ**	82.6 ± 12.0^{a}	88.1 ± 9.2^{a}	36.1 ± 16.5^{b}	73.7 ± 16.8^{a}	78.0 ± 15.5^{a}	73.2 ± 11.4^{a}	**< 0.001**
**CHL**	82.6 ± 12.0^{a}	88.1 ± 9.2^{a}	21.8 ± 6.8^{b}	28.7 ± 14.0^{b}	58.3 ± 15.3^{c}	33.0 ± 9.5^{b}	**< 0.001**
**Prog (%)**							
**5 min**							
**NPX**	70.4 ± 12.4^{a}	78.1 ± 12.2^{a}	52.2 ± 15.1^{b}	46.9 ± 15.9^{b}	41.4 ± 20.8^{b}	22.8 ± 13.3^{c}	**< 0.001**
**DCF**	70.4 ± 12.4^{a}	78.1 ± 12.2^{a}	25.0 ± 15.0^{bc}	18.3 ± 11.4^{b}	14.8 ± 11.3^{bc}	9.1 ± 12.6^{c}	**< 0.001**
**SX**	70.4 ± 12.4^{ac}	78.1 ± 12.2^{a}	60.6 ± 14.6^{c}	56.7 ± 18.0^{c}	14.8 ± 10.4^{d}	10.7 ± 13.3^{d}	**< 0.001**
**ATZ**	70.4 ± 12.4^{a}	78.1 ± 12.2^{a}	19.4 ± 16.2^{b}	44.5 ± 17.4^{c}	55.1 ± 17.7^{c}	53.8 ± 15.1^{c}	**< 0.001**
**CHL**	70.4 ± 12.4^{a}	78.1 ± 12.2^{a}	6.7 ± 3.0^{b}	9.3 ± 6.0^{b}	22.0 ± 13.3^{c}	6.4 ± 2.7^{b}	**< 0.001**
**30 min**							

**NPX**	66.9 ± 11.3^{a}	81.9 ± 8.0^{b}	51.4 ± 13.9^{cd}	57.1 ± 10.3^{ac}	42.0 ± 19.0^{d}	25.9 ± 11.7^{e}	**< 0.001**
**DCF**	66.9 ± 11.3^{a}	81.9 ± 8.0^{b}	25.2 ± 10.3^{c}	17.7 ± 11.8^{c}	16.0 ± 10.6^{c}	6.2 ± 8.7^{d}	**< 0.001**
**SX**	66.9 ± 11.3^{a}	81.9 ± 8.0^{b}	52.8 ± 19.2^{c}	49.4 ± 19.0^{c}	12.4 ± 8.4^{d}	4.1 ± 5.8^{d}	**< 0.001**
**ATZ**	66.9 ± 11.3^{a}	81.9 ± 8.0^{b}	15.6 ± 10.8^{c}	47.1 ± 16.0^{d}	57.5 ± 15.7^{ad}	53.3 ± 8.7^{d}	**< 0.001**
**CHL**	66.9 ± 11.3^{a}	81.9 ± 8.0^{b}	7.0 ± 6.5^{cd}	8.2 ± 5.1^{c}	15.6 ± 7.8^{d}	8.2 ± 4.7^{cd}	**< 0.001**
**60 min**							
**NPX**	60.1 ± 15.6^{a}	76.6 ± 14.1^{b}	43.2 ± 13.3^{c}	44.3 ± 13.6^{c}	36.5 ± 14.3^{cd}	24.4 ± 10.4^{d}	**< 0.001**
**DCF**	60.1 ± 15.6^{a}	76.6 ± 14.1^{b}	26.0 ± 13.4^{c}	19.6 ± 11.1^{c}	12.4 ± 7.8^{c}	10.1 ± 14.9^{c}	**< 0.001**
**SX**	60.1 ± 15.6^{a}	76.6 ± 14.1^{b}	49.1 ± 14.1^{c}	46.2 ± 18.8^{c}	12.4 ± 8.5^{d}	5.7 ± 5.0^{d}	**< 0.001**
**ATZ**	60.1 ± 15.6^{a}	76.6 ± 14.1^{b}	11.4 ± 7.3^{c}	45.8 ± 14.4^{a}	50.1 ± 15.9^{a}	50.7 ± 10.9^{a}	**< 0.001**
**CHL**	60.1 ± 15.6^{a}	76.6 ± 14.1^{b}	4.8 ± 3.0^{c}	5.4 ± 3.5^{c}	19.6 ± 10.6^{d}	7.9 ± 4.9^{c}	**< 0.001**
**Rapid (%)**							
**5 min**							
**NPX**	44.3 ± 20.4^{a}	57.6 ± 14.7^{b}	24.7 ± 9.7^{c}	21.4 ± 10.5^{c}	18.3 ± 16.7^{c}	8.5 ± 7.8^{c}	**< 0.001**
**DCF**	44.3 ± 20.4^{a}	57.6 ± 14.7^{a}	8.4 ± 8.7^{b}	5.0 ± 4.2^{bc}	7.0 ± 7.6^{bc}	3.3 ± 5.1^{c}	**< 0.001**
**SX**	44.3 ± 20.4^{ab}	57.6 ± 14.7^{b}	36.2 ± 20.6^{b}	33.9 ± 23.0^{b}	6.3 ± 4.7^{c}	6.3 ± 10.1^{c}	**< 0.001**
**ATZ**	44.3 ± 20.4^{ab}	57.6 ± 14.7^{b}	10.8 ± 10.4^{c}	21.5 ± 16.0^{dc}	39.0 ± 20.3^{ad}	34.3 ± 19.2^{ad}	**< 0.001**
**CHL**	44.3 ± 20.4^{a}	57.6 ± 14.7^{a}	3.2 ± 1.4^{b}	4.9 ± 3.4^{b}	14.4 ± 11.0^{c}	4.4 ± 2.7^{d}	**< 0.001**
**30 min**							
**NPX**	47.0 ± 19.7^{a}	71.5 ± 9.0^{b}	27.0 ± 13.6^{cd}	33.3 ± 17.9^{c}	23.3 ± 18.5^{cd}	11.0 ± 9.0^{d}	**< 0.001**
**DCF**	47.0 ± 19.7^{a}	71.5 ± 9.0^{a}	10.2 ± 6.5^{b}	5.8 ± 6.1^{cd}	6.8 ± 6.6^{c}	3.5 ± 5.2^{d}	**< 0.001**
**SX**	47.0 ± 19.7^{a}	71.5 ± 9.0^{b}	35.2 ± 21.9^{ac}	29.8 ± 22.0^{c}	6.3 ± 4.7^{d}	2.9 ± 4.2^{d}	**< 0.001**
**ATZ**	47.0 ± 19.7^{a}	71.5 ± 9.0^{b}	10.4 ± 8.3^{c}	28.0 ± 15.1^{d}	36.6 ± 17.8^{ad}	34.3 ± 8.3^{ad}	**< 0.001**
**CHL**	47.0 ± 19.7^{a}	71.5 ± 9.0^{a}	4.3 ± 4.8^{b}	4.6 ± 2.8^{b}	10.7 ± 7.9^{c}	4.7 ± 3.2^{b}	**< 0.001**
**60 min**							
**NPX**	40.7 ± 21.9^{a}	67.6 ± 16.3^{b}	24.5 ± 11.7^{c}	23.7 ± 10.0^{c}	20.2 ± 14.1^{cd}	11.6 ± 7.5^{d}	**< 0.001**
**DCF**	40.7 ± 21.9^{a}	67.6 ± 16.3^{a}	9.7 ± 6.0^{b}	6.1 ± 4.6^{bc}	6.2 ± 4.0^{bc}	5.3 ± 9.4^{c}	**< 0.001**
**SX**	40.7 ± 21.9^{a}	67.6 ± 16.3^{b}	31.3 ± 18.9^{a}	27.8 ± 17.4^{a}	6.6 ± 4.5^{c}	4.5 ± 4.6^{c}	**< 0.001**
**ATZ**	40.7 ± 21.9^{a}	67.6 ± 16.3^{b}	7.7 ± 4.2^{c}	26.0 ± 10.7^{d}	33.2 ± 13.2^{ad}	32.8 ± 8.9^{ad}	**< 0.001**
**CHL**	40.7 ± 21.9^{a}	67.6 ± 16.3^{a}	3.4 ± 2.1^{b}	3.3 ± 2.6^{b}	12.4 ± 7.9^{c}	5.9 ± 3.9^{c}	**< 0.001**
**Medium (%)**							

**5 min**							
**NPX**	31.2 ± 14.7	26.3 ± 9.0[a]	33.9 ± 15.1	34.8 ± 15.5	31.7 ± 11.7	23.9 ± 12.6	0.403
**DCF**	31.2 ± 14.7[a]	26.3 ± 9.0[ab / a]	26.7 ± 9.9[ab]	22.4 ± 11.7[ab]	16.5 ± 11.2[ab]	13.5 ± 12.5[b]	**0.012**
**SX**	31.2 ± 14.7	26.3 ± 9.0[a]	32.1 ± 14.5	29.0 ± 15.9	24.2 ± 7.3	19.1 ± 15.0	0.251
**ATZ**	31.2 ± 14.7	26.3 ± 9.0[a]	16.3 ± 11.1	31.9 ± 11.2	26.0 ± 12.6	27.6 ± 11.5	0.062
**CHL**	31.2 ± 14.7[a]	26.3 ± 9.0[a / a]	9.3 ± 5.1[b]	11.2 ± 5.4[b]	24.4 ± 8.7[a]	8.7 ± 4.0[b]	**< 0.001**
**30 min**							
**NPX**	25.8 ± 15.5[a]	13.4 ± 4.5[b / b]	32.8 ± 15.2[a]	33.6 ± 11.8[a]	28.7 ± 9.7[a]	29.5 ± 10.6[a]	**0.007**
**DCF**	25.8 ± 15.5[a]	13.4 ± 4.5[bc / b]	26.8 ± 10.2[a]	21.1 ± 10.4[ab]	19.6 ± 10.6[ab]	8.9 ± 6.4[c]	**0.003**
**SX**	25.8 ± 15.5	13.4 ± 4.5[b]	24.2 ± 8.5	28.0 ± 12.0	20.0 ± 7.2	10.9 ± 9.6	0.202
**ATZ**	25.8 ± 15.5[a]	13.4 ± 4.5[b / b]	13.4 ± 6.2[b]	30.8 ± 11.4[a]	31.9 ± 14.6[a]	28.2 ± 12.7[a]	**0.001**
**CHL**	25.8 ± 15.5[a]	13.4 ± 4.5[ac / b]	8.9 ± 6.4[b]	9.6 ± 2.8[bc]	19.2 ± 7.5[a]	12.1 ± 6.0[bc]	**0.003**
**60 min**							
**NPX**	25.5 ± 14.6[a]	13.2 ± 5.1[b / b]	26.7 ± 12.6[a]	30.9 ± 11.2[a]	28.1 ± 5.5[a]	27.0 ± 8.6[a]	**0.008**
**DCF**	25.5 ± 14.6[ab]	13.2 ± 5.1[ac / b]	25.3 ± 10.2[b]	24.1 ± 9.2[ab]	16.3 ± 10.9[abc]	11.2 ± 8.6[c]	**0.004**
**SX**	25.5 ± 14.6[a]	13.2 ± 5.1[bc / b]	25.7 ± 10.5[a]	26.0 ± 10.2[a]	18.2 ± 8.9[ab]	8.5 ± 5.2[c]	**< 0.001**
**ATZ**	25.5 ± 14.6[a]	13.2 ± 5.1[b / b]	11.6 ± 7.1[b]	29.8 ± 11.1[a]	29.1 ± 13.3[a]	28.0 ± 11.0[a]	**< 0.001**
**CHL**	25.5 ± 14.6[a]	13.2 ± 5.1[b / b]	6.0 ± 2.6[c]	8.1 ± 4.7[c]	20.3 ± 7.5[a]	9.2 ± 3.5[bc]	**< 0.001**
**Slow (%)**							
**5 min**							
**NPX**	11.3 ± 3.8[ab]	7.5 ± 4.3[b]	18.4 ± 8.7[ac]	19.8 ± 9.9[c]	30.1 ± 15.7[cd]	42.5 ± 18.0[d]	**< 0.001**
**DCF**	11.3 ± 3.8[a]	7.5 ± 4.3[a]	40.9 ± 15.5[b]	42.4 ± 16.3[b]	40.4 ± 20.1[b]	40.1 ± 21.6[b]	**< 0.001**
**SX**	11.3 ± 3.8[a]	7.5 ± 4.3[a]	18.1 ± 8.9[b]	22.4 ± 11.6[b]	42.7 ± 13.4[c]	37.5 ± 18.3[c]	**< 0.001**
**ATZ**	11.3 ± 3.8[a]	7.5 ± 4.3[b]	24.3 ± 16.8[c]	28.1 ± 20.4[c]	16.0 ± 10.5[ac]	14.8 ± 8.1[ac]	**0.008**
**CHL**	11.3 ± 3.8[ab]	7.5 ± 4.3[b]	13.9 ± 10.6[ab]	19.4 ± 13.6[a]	31.4 ± 14.3[c]	17.6 ± 9.2[a]	**< 0.001**
**30 min**							
**NPX**	13.1 ± 5.8[a]	5.8 ± 2.9[b]	17.1 ± 7.4[ac]	16.5 ± 10.2[ac]	25.8 ± 14.5[cd]	34.3 ± 15.4[d]	**< 0.001**
**DCF**	13.1 ± 5.8[a]	5.8 ± 2.9[b]	37.9 ± 14.1[c]	40.4 ± 13.9[c]	38.8 ± 19.3[c]	42.5 ± 22.5[c]	**< 0.001**
**SX**	13.1 ± 5.8[a]	5.8 ± 2.9[b]	21.0 ± 10.2[c]	20.3 ± 10.0[c]	36.8 ± 11.2[d]	31.7 ± 8.6[d]	**< 0.001**
**ATZ**	13.1 ± 5.8[ac]	5.8 ± 2.9[b]	16.4 ± 10.7[ac]	20.6 ± 10.9[c]	14.8 ± 7.4[ac]	11.7 ± 4.3[a]	**0.001**
**CHL**	13.1 ± 5.8[ac]	5.8 ± 2.9[b]	17.1 ± 8.8[ab]	18.3 ± 9.5[ac]	28.5 ± 12.1[c]	20.4 ± 12.0[ac]	**0.003**

**60 min**							
**NPX**	16.4 ± 9.2[a]	7.4 ± 5.5[a]	17.6 ± 8.9[a]	15.6 ± 9.0[a]	28.1 ± 9.6[b]	37.4 ± 12.8[c]	**< 0.001**
**DCF**	16.4 ± 9.2[ac]	7.4 ± 5.5[c]	37.5 ± 10.2[b]	37.9 ± 15.1[b]	28.9 ± 16.2[ab]	41.4 ± 19.2[b]	**< 0.001**
**SX**	16.4 ± 9.2a	7.4 ± 5.5[b]	23.0 ± 7.5[ac]	21.3 ± 8.2[ac]	36.1 ± 10.7[d]	30.4 ± 12.1[dc]	**< 0.001**
**ATZ**	16.4 ± 9.2	7.4 ± 5.5	16.7 ± 11.4	17.8 ± 7.4	15.8 ± 8.4	12.5 ± 4.4	0.053
**CHL**	16.4 ± 9.2[ac]	7.4 ± 5.5[b]	12.5 ± 6.2[ab]	17.3 ± 9.5[ac]	25.5 ± 9.8[c]	17.8 ± 7.9[a]	**< 0.001**
**RP (%)**							
**5 min**							
**NPX**	23.2 ± 11.3[a]	25.3 ± 9.6[a]	10.0 ± 6.3[b]	9.2 ± 5.7[b]	7.1 ± 7.7[bc]	2.7 ± 3.4[c]	**< 0.001**
**DCF**	23.2 ± 11.3[a]	25.3 ± 9.6[a]	3.7 ± 4.7[b]	1.7 ± 1.6[bc]	3.1 ± 4.5[bc]	1.2 ± 2.1[c]	**< 0.001**
**SX**	23.2 ± 11.3[a]	25.3 ± 9.6[a]	17.7 ± 8.7[ab]	14.1 ± 8.1[b]	1.9 ± 3.4[c]	0.9 ± 1.5[c]	**< 0.001**
**ATZ**	23.2 ± 11.3[a]	25.3 ± 9.6[a]	4.2 ± 4.7[b]	8.5 ± 9.0[b]	10.4 ± 9.3[b]	13.4 ± 9.1[b]	**< 0.001**
**CHL**	23.2 ± 11.3[a]	25.3 ± 9.6[a]	1.6 ± 1.5[bc]	1.2 ± 0.9[bc]	5.9 ± 8.0[b]	1.0 ± 0.8[c]	**< 0.001**
**30 min**							
**NPX**	26.0 ± 11.1[a]	35.3 ± 10.6[a]	14.1 ± 7.4[b]	12.4 ± 6.5[b]	9.0 ± 8.6[b]	3.1 ± 2.8[c]	**< 0.001**
**DCF**	26.0 ± 11.1[a]	35.3 ± 10.6[a]	4.0 ± 3.4[b]	1.9 ± 3.3[c]	2.1 ± 2.6[c]	1.2 ± 2.5[c]	**< 0.001**
**SX**	26.0 ± 11.1[ab]	35.3 ± 10.6[a]	20.8 ± 16.1[ac]	11.7 ± 9.8[d]	1.9 ± 2.7[e]	0.5 ± 1.7[e]	**< 0.001**
**ATZ**	26.0 ± 11.1[a]	35.3 ± 10.6[a]	3.7 ± 4.3[b]	11.5 ± 7.7[c]	10.6 ± 8.3[c]	12.4 ± 4.1[c]	**< 0.001**
**CHL**	26.0 ± 11.1[a]	35.3 ± 10.6[a]	1.9 ± 2.7[b]	1.6 ± 1.9[b]	3.4 ± 4.6[b]	1.4 ± 1.6[b]	**< 0.001**
**60 min**							
**NPX**	21.8 ± 11.7[a]	34.1 ± 10.6[b]	11.1 ± 4.4[d]	7.9 ± 5.3[de]	7.0 ± 4.9[e]	2.7 ± 2.2[c]	**< 0.001**
**DCF**	21.8 ± 11.7[a]	34.1 ± 10.6[a]	3.7 ± 2.8[b]	2.5 ± 2.8[bc]	2.4 ± 1.7[b]	2.4 ± 5.1[c]	**< 0.001**
**SX**	21.8 ± 11.7[a]	34.1 ± 10.6[b]	18.0 ± 13.0[ac]	12.9 ± 8.4[c]	1.5 ± 1.7[d]	1.2 ± 2.3[d]	**< 0.001**
**ATZ**	21.8 ± 11.7[a]	34.1 ± 10.6[b]	2.2 ± 1.3[c]	10.7 ± 7.4[d]	10.0 ± 7.4[d]	12.6 ± 6.4[d]	**< 0.001**
**CHL**	21.8 ± 11.7[a]	34.1 ± 10.6[a]	1.1 ± 0.8[b]	0.7 ± 0.9[b]	3.3 ± 2.8[c]	1.5 ± 2.0[b]	**< 0.001**
**MP (%)**							
**5 min**							
**NPX**	47.2 ± 8.3[ab]	52.8 ± 10.6[b]	42.2 ± 10.7[ab]	37.7 ± 12.6[a]	34.3 ± 14.2[a]	20.1 ± 11.5[c]	**< 0.001**
**DCF**	47.2 ± 8.3[a]	52.8 ± 10.6[a]	21.3 ± 11.0[b]	16.6 ± 11.1[bc]	11.7 ± 7.9[bc]	7.9 ± 10.5[c]	**< 0.001**
**SX**	47.2 ± 8.3[a]	52.8 ± 10.6[a]	42.9 ± 11.0[a]	42.5 ± 12.0[a]	13.0 ± 7.7[b]	9.9 ± 11.9[b]	**< 0.001**
**ATZ**	47.2 ± 8.3[ac]	52.8 ± 10.6[a]	15.1 ± 13.7[b]	36.0 ± 10.8[c]	44.7 ± 11.0[ac]	40.3 ± 10.2[ac]	**< 0.001**

**CHL**	47.2 ± 8.3^{a}	52.8 ± 10.6^{a}	5.1 ± 2.5^{b}	8.1 ± 5.2^{b}	16.1 ± 7.6^{c}	5.4 ± 2.2^{b}	**< 0.001**
**30 min**							
**NPX**	40.9 ± 7.1^{ab}	46.5 ± 12.8^{a}	37.3 ± 10.7^{ab}	44.7 ± 4.9^{a}	33.0 ± 11.6^{b}	22.8 ± 9.3^{c}	**< 0.001**
**DCF**	40.9 ± 7.1^{a}	46.5 ± 12.8^{a}	21.2 ± 7.4^{b}	15.8 ± 9.4^{b}	13.9 ± 8.5^{b}	5.0 ± 6.3^{c}	**< 0.001**
**SX**	40.9 ± 7.1^{ab}	46.5 ± 12.8^{a}	32.1 ± 7.1^{b}	37.7 ± 11.0^{ab}	10.6 ± 7.1^{c}	3.5 ± 4.2^{c}	**< 0.001**
**ATZ**	40.9 ± 7.1^{a}	46.5 ± 12.8^{a}	11.9 ± 7.0^{b}	35.6 ± 10.1^{a}	46.9 ± 11.3^{a}	41.0 ± 8.4^{a}	**< 0.001**
**CHL**	40.9 ± 7.1^{a}	46.5 ± 12.8^{a}	5.2 ± 4.1^{b}	6.6 ± 3.4^{b}	12.2 ± 4.6^{c}	6.8 ± 3.8^{b}	**< 0.001**
**60 min**							
**NPX**	38.3 ± 10.4^{ab}	42.5 ± 9.3^{b}	32.1 ± 11.4^{abc}	36.4 ± 9.7^{ab}	29.5 ± 9.8^{ac}	21.7 ± 8.8^{c}	**< 0.001**
**DCF**	38.3 ± 10.4^{a}	42.5 ± 9.3^{a}	22.2 ± 10.8^{b}	17.1 ± 8.6b^{c}	10.0 ± 6.6^{c}	7.7 ± 9.9^{c}	**< 0.001**
**SX**	38.3 ± 10.4^{ab}	42.5 ± 9.3^{a}	31.0 ± 8.0^{b}	33.3 ± 11.7^{ab}	10.9 ± 7.9^{c}	4.5 ± 3.8^{c}	**< 0.001**
**ATZ**	38.3 ± 10.4^{a}	42.5 ± 9.3^{a}	9.2 ± 6.7^{b}	35.1 ± 11.0^{a}	40.2 ± 10.5^{a}	38.2 ± 8.9^{a}	**< 0.001**
**CHL**	38.3 ± 10.4^{a}	42.5 ± 9.3^{a}	3.7 ± 2.4^{b}	4.7 ± 2.8^{b}	16.3 ± 8.9^{b}	6.3 ± 3.1^{b}	**< 0.001**
**NP (%)**							
**5 min**							
**NPX**	16.4 ± 5.9^{a}	13.3 ± 9.1^{a}	24.8 ± 10.3^{bc}	29.1 ± 9.4^{c}	38.8 ± 14.0^{dc}	52.2 ± 14.9^{d}	**< 0.001**
**DCF**	16.4 ± 5.9^{a}	13.3 ± 9.1^{a}	51.0 ± 16.9^{b}	51.4 ± 16.4^{b}	49.1 ± 21.3^{b}	47.8 ± 21.3^{b}	**< 0.001**
**SX**	16.4 ± 5.9^{a}	13.3 ± 9.1^{a}	25.7 ± 12.3^{b}	28.6 ± 12.0^{b}	58.3 ± 14.8^{c}	52.1 ± 21.0^{c}	**< 0.001**
**ATZ**	16.4 ± 5.9^{ab}	13.3 ± 9.1^{b}	32.1 ± 16.6^{c}	37.0 ± 20.4^{c}	25.9 ± 12.4^{c}	22.9 ± 10.1^{ac}	**0.006**
**CHL**	16.4 ± 5.9^{ab}	13.3 ± 9.1^{b}	19.6 ± 13.6^{ab}	26.2 ± 15.5^{a}	48.2 ± 17.2^{c}	24.3 ± 11.6^{a}	**< 0.001**
**30 min**							
**NPX**	19.0 ± 8.6^{a}	8.9 ± 3.7^{b}	25.5 ± 10.3^{a}	26.3 ± 11.2^{a}	35.8 ± 13.8^{c}	48.9 ± 10.0^{d}	**< 0.001**
**DCF**	19.0 ± 8.6^{a}	8.9 ± 3.7^{a}	49.7 ± 12.6^{b}	49.5 ± 14.8^{b}	49.2 ± 19.7^{b}	48.7 ± 23.1^{b}	**< 0.001**
**SX**	19.0 ± 8.6^{ac}	8.9 ± 3.7^{b}	27.5 ± 12.0^{c}	28.7 ± 10.6^{c}	50.6 ± 13.6^{d}	41.5 ± 14.5^{cd}	**< 0.001**
**ATZ**	19.0 ± 8.6^{a}	8.9 ± 3.7^{b}	24.6 ± 11.3^{ac}	32.4 ± 12.8^{c}	25.8 ± 9.6^{ac}	20.9 ± 6.9^{a}	**< 0.001**
**CHL**	19.0 ± 8.6^{a}	8.9 ± 3.7^{b}	23.2 ± 13.1^{a}	24.4 ± 10.5^{a}	42.7 ± 16.8^{c}	29.0 ± 14.2^{ac}	**< 0.001**
**60 min**							
**NPX**	22.4 ± 11.9^{a}	11.5 ± 7.2^{b}	25.6 ± 11.3^{a}	25.9 ± 12.7^{a}	40.0 ± 7.8^{c}	51.5 ± 11.4^{d}	**< 0.001**
**DCF**	22.4 ± 11.9^{a}	11.5 ± 7.2^{b}	46.5 ± 9.5^{b}	48.5 ± 17.7^{b}	39.0 ± 20.9^{b}	47.8 ± 20.4^{b}	**< 0.001**
**SX**	22.4 ± 11.9^{a}	11.5 ± 7.2^{b}	30.9 ± 9.3^{ad}	29.0 ± 10.2^{ad}	48.6 ± 13.2^{c}	37.7 ± 14.5^{d}	**< 0.001**
**ATZ**	22.4 ± 11.9^{a}	11.5 ± 7.2^{b}	24.7 ± 12.5^{a}	27.9 ± 11.2^{a}	27.9 ± 10.1^{a}	22.5 ± 4.8^{a}	**0.006**

**CHL**	22.4 ± 11.9[a]	11.5 ± 7.2[b]	17.0 ± 7.0[ab]	23.3 ± 12.2[a]	38.7 ± 13.9[c]	25.1 ± 10.3[a]	**< 0.001**

Note: T1 - T4 is equal to the ascending concentrations of each individual treatment. Values labelled with different letters (a, b, c, d, e) were significantly different between concentrations levels for individual treatments ($p < 0.05$). Values in the same column labelled in red with different letters (a, b, c) were significantly different between time points for individual concentration levels for an individual treatment ($p < 0.05$). *ATZ* , atrazine; *CAP* , capacitation HTF; *CHL* , chlorpyrifos; *DCF* , diclofenac; *min* , minutes; *MP* , medium progressive; *NP* , non-progressive; *NPX* , naproxen; *Prog* , progressive; *RP* , rapid progressive; *SD* , standard deviation; *SX* , sulfamethoxazole; *T* , treatment; *Total Mot* , total motility.

Table S3: Average kinematic parameters of human spermatozoa after 5-, 30- and 60-minutes exposure to different concentrations of naproxen, diclofenac, sulfamethoxazole, atrazine and chlorpyrifos (n=10) (mean ± SD).

	Treatment						ANOVA
	Control	CAP	T1	T2	T3	T4	
**VCL (µm/s)**							
**5 min**							
**NPX**	102.2 ± 15.5^{a}	115.0 ± 12.3$^{b / a}$	90.6 ± 11.1^{c}	87.8 ± 11.6^{c}	80.9 ± 12.6^{cd}	69.9 ± 9.8^{d}	**< 0.001**
**DCF**	102.2 ± 15.5^{a}	115.0 ± 12.3$^{b / a}$	71.0 ± 8.1^{c}	66.2 ± 7.2^{c}	67.1 ± 10.6^{c}	60.0 ± 12.3^{c}	**< 0.001**
**SX**	102.2 ± 15.5^{ac}	115.0 ± 12.3$^{ab / a}$	96.7 ± 17.3^{c}	93.7 ± 18.5^{c}	68.4 ± 7.9^{d}	63.9 ± 11.5^{d}	**< 0.001**
**ATZ**	102.2 ± 15.5^{ac}	115.0 ± 12.3$^{b / a}$	76.9 ± 15.1^{b}	84.5 ± 14.0^{bc}	99.3 ± 15.6^{c}	95.9 ± 14.2^{c}	**< 0.001**
**CHL**	102.2 ± 15.5^{a}	115.0 ± 12.3$^{b / a}$	73.6 ± 10.4^{c}	72.0 ± 12.9^{c}	77.0 ± 11.6^{c}	70.6 ± 9.4^{c}	**< 0.001**
**30 min**							
**NPX**	107.5 ± 18.6^{a}	130.6 ± 7.2$^{b / b}$	91.8 ± 10.4^{c}	94.7 ± 13.4^{c}	85.7 ± 14.7^{cd}	74.5 ± 9.4^{d}	**< 0.001**
**DCF**	107.5 ± 18.6^{a}	130.6 ± 7.2$^{a / b}$	72.7 ± 7.3^{b}	67.6 ± 8.1b^{c}	67.7 ± 10.6^{bc}	60.4 ± 13.4^{c}	**< 0.001**
**SX**	107.5 ± 18.6^{a}	130.6 ± 7.2$^{b / b}$	93.6 ± 17.2^{c}	90.9 ± 15.4^{c}	68.2 ± 7.7^{d}	59.8 ± 8.9^{d}	**< 0.001**
**ATZ**	107.5 ± 18.6^{a}	130.6 ± 7.2$^{b / b}$	78.3 ± 14.8^{c}	88.8 ± 12.7^{cd}	95.9 ± 14.2^{ad}	98.5 ± 6.8^{ad}	**< 0.001**
**CHL**	107.5 ± 18.6^{a}	130.6 ± 7.2$^{a / b}$	69.1 ± 8.2^{b}	71.2 ± 8.2^{b}	75.3 ± 10.6^{b}	70.5 ± 9.6^{b}	**< 0.001**
**60 min**							
**NPX**	105.8 ± 25.0^{a}	128.9 ± 18.8$^{b / ab}$	91.5 ± 9.9^{a}	90.7 ± 10.3^{a}	81.5 ± 11.5^{c}	73.7 ± 8.4^{c}	**< 0.001**
**DCF**	105.8 ± 25.0^{a}	128.9 ± 18.8$^{a / ab}$	71.6 ± 7.3^{b}	69.3 ± 6.8^{b}	71.2 ± 11.6^{b}	60.2 ± 14.0^{c}	**< 0.001**
**SX**	105.8 ± 25.0^{ac}	128.9 ± 18.8$^{b / ab}$	91.7 ± 13.8^{c}	88.6 ± 13.4^{c}	67.5 ± 7.8^{d}	62.3 ± 6.4^{d}	**< 0.001**
**ATZ**	105.8 ± 25.0^{a}	128.9 ± 18.8$^{b / ab}$	76.8 ± 9.3^{c}	90.1 ± 8.3^{a}	96.1 ± 13.1^{a}	96.6 ± 7.3^{a}	**< 0.001**
**CHL**	105.8 ± 25.0^{a}	128.9 ± 18.8$^{a / ab}$	72.0 ± 10.9^{bc}	67.5 ± 10.1^{c}	76.5 ± 8.1^{b}	72.5 ± 9.1^{bc}	**< 0.001**
**VAP (µm/s)**							
**5 min**							
**NPX**	56.4 ± 5.7^{ab}	59.8 ± 9.0^{b}	51.9 ± 7.8^{ac}	49.8 ± 6.4^{ac}	45.7 ± 7.0^{cd}	39.7 ± 5.5^{d}	**< 0.001**
**DCF**	56.4 ± 5.7^{a}	59.8 ± 9.0^{a}	39.9 ± 4.1^{b}	37.5 ± 4.4^{b}	37.4 ± 5.1^{b}	34.3 ± 5.4^{b}	**< 0.001**
**SX**	56.4 ± 5.7^{a}	59.8 ± 9.0^{a}	54.2 ± 7.2^{ab}	50.4 ± 6.4^{b}	39.7 ± 2.9^{c}	38.7 ± 4.2^{c}	**< 0.001**
**ATZ**	56.4 ± 5.7^{a}	59.8 ± 9.0^{a}	42.3 ± 5.4^{b}	46.5 ± 5.4^{b}	48.7 ± 7.0^{b}	49.9 ± 6.5^{b}	**< 0.001**
**CHL**	56.4 ± 5.7^{a}	59.8 ± 9.0^{a}	41.8 ± 4.3^{b}	41.2 ± 5.1^{b}	42.9 ± 4.5^{b}	40.2 ± 3.5^{b}	**< 0.001**
**30 min**							

**NPX**	59.3 ± 4.9^{a}	65.5 ± 5.8^{b}	52.8 ± 5.0^{cd}	52.6 ± 4.5^{c}	47.8 ± 5.9^{d}	42.6 ± 4.8^{e}	**< 0.001**
**DCF**	59.3 ± 4.9^{a}	65.5 ± 5.8^{b}	40.5 ± 2.9^{c}	38.2 ± 4.2^{c}	38.3 ± 3.4^{c}	35.7 ± 6.1^{c}	**< 0.001**
**SX**	59.3 ± 4.9^{a}	65.5 ± 5.8^{b}	52.1 ± 7.1^{c}	50.1 ± 6.4^{c}	39.8 ± 3.2^{d}	37.8 ± 3.4^{d}	**< 0.001**
**ATZ**	59.3 ± 4.9^{a}	65.5 ± 5.8^{b}	42.5 ± 5.2^{c}	47.2 ± 4.6^{d}	48.7 ± 5.3^{d}	50.8 ± 4.6^{d}	**< 0.001**
**CHL**	59.3 ± 4.9^{a}	65.5 ± 5.8^{b}	39.3 ± 3.4^{c}	41 ± 3.3^{c}	42.7 ± 3.7^{c}	39.7 ± 4.2^{c}	**< 0.001**
**60 min**							
**NPX**	58.4 ± 9.1^{a}	65.0 ± 9.9^{a}	52.4 ± 2.9^{b}	50.2 ± 3.3^{b}	45.7 ± 4.6^{c}	41.9 ± 3.8^{c}	**< 0.001**
**DCF**	58.4 ± 9.1^{a}	65.0 ± 9.9^{a}	41.4 ± 4.2^{b}	39.9 ± 3.5^{bc}	40.3 ± 4.0^{b}	36.1 ± 6.7^{c}	**< 0.001**
**SX**	58.4 ± 9.1^{a}	65.0 ± 9.9^{a}	51.5 ± 6.1^{b}	49.9 ± 6.1^{b}	40.2 ± 2.7^{c}	37.7 ± 2.2^{c}	**< 0.001**
**ATZ**	58.4 ± 9.1^{a}	65.0 ± 9.9^{a}	42.2 ± 3.4^{b}	49.0 ± 4.4^{c}	49.1 ± 5.3^{c}	51.9 ± 3.5^{c}	**< 0.001**
**CHL**	58.4 ± 9.1^{a}	65.0 ± 9.9^{a}	40.3 ± 4.5^{b}	38.6 ± 3.8^{b}	42.5 ± 2.4^{b}	39.9 ± 3.8^{b}	**< 0.001**
**VSL (μm/s)**							
**5 min**							
**NPX**	45.6 ± 8.1^{a}	46.0 ± 11.4^{a}	41.1 ± 9.9^{a}	38.2 ± 8.6^{ab}	36.1 ± 8.5^{ab}	29.5 ± 7.2^{b}	**0.001**
**DCF**	45.6 ± 8.1^{a}	46.0 ± 11.4^{a}	30.6 ± 5.2^{b}	27.5 ± 5.4^{b}	26.1 ± 5.1^{bc}	23.2 ± 4.8^{c}	**< 0.001**
**SX**	45.6 ± 8.1^{a}	46.0 ± 11.4^{a}	43.6 ± 7.6^{a}	39.1 ± 6.9^{a}	25.2 ± 4.8^{b}	22.0 ± 4.3^{b}	**< 0.001**
**ATZ**	45.6 ± 8.1^{a}	46.0 ± 11.4^{b}	29.2 ± 6.4^{c}	35.6 ± 7.0^{c}	32.6 ± 8.2^{c}	37.8 ± 5.5^{bc}	**< 0.001**
**CHL**	45.6 ± 8.1^{a}	46.0 ± 11.4^{a}	26.9 ± 6.6^{b}	24.9 ± 6.4^{bc}	27.3 ± 6.1^{b}	22.0 ± 3.4^{c}	**< 0.001**
**30 min**							
**NPX**	47.0 ± 4.4^{a}	48.8 ± 7.9^{a}	41.3 ± 6.2^{b}	38.9 ± 5.3^{b}	35.4 ± 5.4^{bc}	31.3 ± 3.4^{c}	**< 0.001**
**DCF**	47.0 ± 4.4^{a}	48.8 ± 7.9^{a}	30.5 ± 3.7^{b}	27.7 ± 4.7^{b}	26.1 ± 3.1^{bc}	22.0 ± 5.4^{c}	**< 0.001**
**SX**	47.0 ± 4.4^{a}	48.8 ± 7.9^{a}	41.2 ± 7.8^{b}	37.8 ± 6.6^{b}	25.2 ± 4.3^{c}	20.4 ± 2.8^{c}	**< 0.001**
**ATZ**	47.0 ± 4.4	48.8 ± 7.9	27.4 ± 5.3	34.3 ± 5.7	34.7 ± 7.4	36.8 ± 6.8	0.068
**CHL**	47.0 ± 4.4^{a}	48.8 ± 7.9^{a}	24.4 ± 6.4^{b}	24.4 ± 6.6^{b}	25.5 ± 5.6^{b}	23.6 ± 4.4^{b}	**< 0.001**
**60 min**							
**NPX**	46.4 ± 8.5^{abc}	48.9 ± 8.4^{b}	40.4 ± 3.8^{c}	36.9 ± 4.5^{d}	33.8 ± 2.5^{e}	29.3 ± 3.1^{f}	**< 0.001**
**DCF**	46.4 ± 8.5^{a}	48.9 ± 8.4^{a}	31.0 ± 4.6^{b}	29.4 ± 3.7^{bc}	26.9 ± 2.9^{c}	22.3 ± 7.6^{d}	**< 0.001**
**SX**	46.4 ± 8.5^{a}	48.9 ± 8.4^{a}	39.6 ± 5.6^{b}	37.5 ± 6.1^{b}	24.9 ± 5.0^{c}	20.7 ± 2.9^{c}	**< 0.001**
**ATZ**	46.4 ± 8.5^{a}	48.9 ± 8.4^{a}	27.0 ± 3.4^{b}	36.1 ± 5.5^{c}	33.8 ± 6.6^{c}	37.6 ± 5.8^{c}	**< 0.001**
**CHL**	46.4 ± 8.5^{a}	48.9 ± 8.4^{a}	25.2 ± 5.2^{b}	20.6 ± 4.9^{b}	26.0 ± 4.6^{b}	22.4 ± 6.5^{b}	**< 0.001**
**STR (%)**							

**5 min**							
**NPX**	79.2 ± 7.7	75.1 ± 9.6	77.0 ± 8.3	74.1 ± 11.1	76.7 ± 8.7	72.2 ± 10.0	0.624
**DCF**	79.2 ± 7.7^{a}	75.1 ± 9.6^{ab}	74.5 ± 6.5^{ab}	70.7 ± 6.9^{abc}	66.5 ± 7.9^{bc}	65.2 ± 5.0^{c}	**< 0.001**
**SX**	79.2 ± 7.7^{a}	75.1 ± 9.6^{a}	78.2 ± 5.5^{a}	75.2 ± 8.5^{a}	62.1 ± 8.1^{b}	55.5 ± 7.8^{b}	**< 0.001**
**ATZ**	79.2 ± 7.7^{a}	75.1 ± 9.6^{ab}	66.2 ± 8.2^{b}	74.5 ± 9.8^{ab}	65.8 ± 11.1^{b}	74.1 ± 5.7^{ab}	**0.007**
**CHL**	79.2 ± 7.7^{a}	75.1 ± 9.6^{a}	61.0 ± 8.9^{b}	58.0 ± 8.1^{b}	62.0 ± 9.0^{b}	53.2 ± 4.2^{b}	**< 0.001**
**30 min**							
**NPX**	76.9 ± 5.2	73.2 ± 6.1	75.7 ± 5.6	72.4 ± 6.7	72.6 ± 7.3	72.3 ± 6.3	0.420
**DCF**	76.9 ± 5.2^{a}	73.2 ± 6.1^{ab}	72.8 ± 4.8^{ab}	70.0 ± 5.9^{bc}	66.0 ± 3.9^{c}	60.1 ± 6.2^{d}	**< 0.001**
**SX**	76.9 ± 5.2^{a}	73.2 ± 6.1^{a}	75.2 ± 5.0^{a}	72.9 ± 6.7^{a}	62.0 ± 6.2^{b}	53.4 ± 4.9^{b}	**< 0.001**
**ATZ**	76.9 ± 5.2^{a}	73.2 ± 6.1^{a}	62.2 ± 7.1^{b}	70.4 ± 6.3^{a}	69.7 ± 8.8^{a}	70.0 ± 7.1^{a}	**0.001**
**CHL**	76.9 ± 5.2^{a}	73.2 ± 6.1^{a}	59.2 ± 9.6^{b}	57.2 ± 11.0^{b}	57.8 ± 10.5^{b}	57.7 ± 5.5^{b}	**< 0.001**
**60 min**							
**NPX**	76.3 ± 5.5^{a}	73.9 ± 4.7^{ab}	74.3 ± 4.6^{ab}	70.8 ± 5.2^{ab}	72.0 ± 5.1^{ab}	68.5 ± 7.0^{b}	**0.035**
**DCF**	76.3 ± 5.5^{a}	73.9 ± 4.7^{a}	72.0 ± 5.6^{a}	70.8 ± 4.0^{a}	64.3 ± 3.8^{b}	59.0 ± 8.6^{c}	**< 0.001**
**SX**	76.3 ± 5.5^{a}	73.9 ± 4.7^{a}	73.4 ± 4.2^{a}	72.0 ± 5.4^{a}	60.2 ± 8.5^{b}	53.5 ± 4.7^{b}	**< 0.001**
**ATZ**	76.3 ± 5.5^{a}	73.9 ± 4.7^{ac}	61.4 ± 5.2^{b}	70.9 ± 5.4^{ac}	66.9 ± 8.0^{c}	70.8 ± 6.6^{ac}	**< 0.001**
**CHL**	76.3 ± 5.5^{a}	73.9 ± 4.7^{a}	60.1 ± 5.9^{b}	51.9 ± 9.0^{b}	59.4 ± 8.3^{b}	53.7 ± 9.7^{b}	**< 0.001**
**LIN (%)**							
**5 min**							
**NPX**	47.7 ± 11.6	43.7 ± 12.4	47.9 ± 12.4	45.5 ± 12.5	46.7 ± 11.4	43.5 ± 10.1	0.926
**DCF**	47.7 ± 11.6	43.7 ± 12.4	44.3 ± 8.2	41.9 ± 7.0	38.9 ± 7.9	38.8 ± 5.1	0.225
**SX**	47.7 ± 11.6^{a}	43.7 ± 12.4^{ab}	47.5 ± 9.6^{a}	44.6 ± 12.1^{ab}	37.1 ± 5.6^{bc}	34.5 ± 5.7^{c}	**0.020**
**ATZ**	47.7 ± 11.6	43.7 ± 12.4	38.4 ± 7.5	44.2 ± 10.6	36.0 ± 10.4	41.9 ± 7.3	0.139
**CHL**	47.7 ± 11.6^{a}	43.7 ± 12.4^{ab}	35.3 ± 5.0^{b}	33.9 ± 4.5^{b}	35.5 ± 5.6^{b}	31.0 ± 2.2^{bc}	**0.002**
**30 min**							
**NPX**	45.9 ± 7.8	40.0 ± 6.5	46.5 ± 8.8	43.5 ± 8.9	43.6 ± 8.4	43.2 ± 6.8	0.525
**DCF**	45.9 ± 7.8^{a}	40.0 ± 6.5^{ab}	43.0 ± 6.7^{ab}	41.5 ± 5.8^{ab}	38.8 ± 5.1^{ab}	36.8 ± 3.5^{b}	**0.025**
**SX**	45.9 ± 7.8^{a}	40.0 ± 6.5^{ab}	44.5 ± 4.8^{a}	43.4 ± 8.8^{a}	36.9 ± 3.6^{bc}	34.3 ± 2.6^{c}	**< 0.001**
**ATZ**	45.9 ± 7.8	40.0 ± 6.5	35.2 ± 5.3	39.8 ± 6.8	38.5 ± 10.0	39.1 ± 8.9	0.085
**CHL**	45.9 ± 7.8^{a}	40.0 ± 6.5^{ab}	34.5 ± 5.6^{b}	33.7 ± 6.7^{b}	33.9 ± 7.1^{b}	33.4 ± 3.0^{b}	**< 0.001**

**60 min**							
**NPX**	45.6 ± 8.1	40.2 ± 4.1	44.9 ± 6.9	42.1 ± 6.4	42.8 ± 5.8	41.1 ± 6.4	0.365
**DCF**	45.6 ± 8.1^{a}	40.2 ± 4.1^{abc}	43.6 ± 5.8^{ab}	42.5 ± 3.8^{ab}	37.8 ± 4.2^{bc}	36.2 ± 4.5^{c}	**0.002**
**SX**	45.6 ± 8.1^{a}	40.2 ± 4.1^{ab}	43.6 ± 4.5^{a}	43.0 ± 6.0^{a}	36.6 ± 4.7^{bc}	33.0 ± 3.6^{c}	**< 0.001**
**ATZ**	45.6 ± 8.1^{a}	40.2 ± 4.1^{ab}	35.2 ± 4.8^{b}	40.7 ± 6.1^{ab}	37.0 ± 8.2^{b}	40.6 ± 7.5^{ab}	**0.021**
**CHL**	45.6 ± 8.1^{a}	40.2 ± 4.1^{b}	34.8 ± 4.1^{c}	30.3 ± 5.9^{c}	33.9 ± 5.2^{bc}	30.6 ± 6.7^{c}	**< 0.001**
**WOB (%)**							
**5 min**							
**NPX**	57.8 ± 9.1	54.8 ± 9.5	59.4 ± 9.7	58.3 ± 8.4	58.3 ± 8.4	58.3 ± 5.9	0.889
**DCF**	57.8 ± 9.1	54.8 ± 9.5	57.6 ± 5.9	57.7 ± 4.3	56.7 ± 5.4	58.6 ± 4.7	0.979
**SX**	57.8 ± 9.1	54.8 ± 9.5	58.4 ± 7.8	56.7 ± 9.8	59.4 ± 3.7	62.0 ± 4.5	0.444
**ATZ**	57.8 ± 9.1	54.8 ± 9.5	56.6 ± 6.2	57.0 ± 7.3	51.4 ± 6.6	54.1 ± 5.4	0.420
**CHL**	57.8 ± 9.1	54.8 ± 9.5	57.9 ± 3.6	58.5 ± 3.3	57.2 ± 3.9	58.7 ± 3.9	0.876
**30 min**							
**NPX**	57.5 ± 6.4	52.5 ± 4.7	58.9 ± 7.2	57.7 ± 6.8	57.9 ± 5.8	58.5 ± 4.7	0.194
**DCF**	57.5 ± 6.4^{ab}	52.5 ± 4.7^{a}	57.2 ± 5.6^{b}	57.8 ± 3.8^{ab}	57.9 ± 5.8^{ab}	60.7 ± 3.8^{b}	**0.030**
**SX**	57.5 ± 6.4^{ab}	52.5 ± 4.7^{a}	57.4 ± 4.6^{ab}	57.3 ± 6.5^{a}	59.6 ± 2.5^{a}	64.7 ± 4.5^{c}	**< 0.001**
**ATZ**	57.5 ± 6.4	52.5 ± 4.7	56.0 ± 4.7	54.5 ± 5.2	52.8 ± 7.1	53.1 ± 6.7	0.332
**CHL**	57.5 ± 6.4^{a}	52.5 ± 4.7^{b}	58.1 ± 3.0^{a}	58.8 ± 3.0^{a}	58.3 ± 4.7^{a}	57.7 ± 2.4^{a}	**0.046**
**60 min**							
**NPX**	57.6 ± 7.2	52.6 ± 3.3	58.4 ± 5.6	57.2 ± 5.5	57.7 ± 4.2	58.4 ± 3.6	0.120
**DCF**	57.6 ± 7.2^{a}	52.6 ± 3.3^{b}	58.9 ± 3.9^{a}	58.6 ± 3.1^{a}	58.3 ± 4.8^{a}	61.3 ± 3.5^{a}	**0.004**
**SX**	57.6 ± 7.2^{a}	52.6 ± 3.3^{b}	57.6 ± 3.5^{a}	57.8 ± 4.5^{a}	60.8 ± 4.0^{ac}	62.4 ± 2.8^{c}	**< 0.001**
**ATZ**	57.6 ± 7.2	52.6 ± 3.3	56.4 ± 3.5	55.4 ± 4.6	52.9 ± 5.5	55.2 ± 5.1	0.212
**CHL**	57.6 ± 7.2	52.6 ± 3.3	57.7 ± 3.6	58.2 ± 3.8	57.1 ± 3.5	56.6 ± 4.4	0.085
**ALH (μm)**							
**5 min**							
**NPX**	2.8 ± 0.6^{ab}	3.2 ± 0.5$^{a\ /a}$	2.5 ± 0.5^{b}	2.5 ± 0.6^{b}	2.3 ± 0.5^{b}	2.2 ± 0.3^{b}	**0.001**
**DCF**	2.8 ± 0.6^{a}	3.2 ± 0.5$^{a\ /a}$	2.2 ± 0.3^{b}	2.1 ± 0.2^{b}	2.1 ± 0.3^{b}	2.0 ± 0.4^{b}	**< 0.001**
**SX**	2.8 ± 0.6^{ab}	3.2 ± 0.5$^{a\ /a}$	2.7 ± 0.7^{ab}	2.7 ± 0.7^{ab}	2.2 ± 0.2^{b}	2.1 ± 0.4^{b}	**0.001**
**ATZ**	2.8 ± 0.6^{ab}	3.2 ± 0.5$^{a\ /a}$	2.4 ± 0.5^{b}	2.6 ± 0.5^{ab}	3.0 ± 0.6^{ab}	2.8 ± 0.5^{ab}	**0.042**

**CHL**	2.8 ± 0.6[a]	3.2 ± 0.5[b /a]	2.4 ± 0.3[a]	2.4 ± 0.4[a]	2.5 ± 0.4[a]	2.3 ± 0.3[a]	**0.001**
**30 min**							
**NPX**	3.0 ± 0.7[a]	3.8 ± 0.3[b / b]	2.6 ± 0.4[a]	2.8 ± 0.5[a]	2.6 ± 0.5[a]	2.3 ± 0.3[a]	**0.001**
**DCF**	3.0 ± 0.7[a]	3.8 ± 0.3[b / b]	2.3 ± 0.3[c]	2.1 ± 0.3[c]	2.2 ± 0.3[c]	2.0 ± 0.4[c]	**< 0.001**
**SX**	3.0 ± 0.7[a]	3.8 ± 0.3[b / b]	2.7 ± 0.5[a]	2.7 ± 0.5[ac]	2.2 ± 0.2[cd]	2.0 ± 0.3[d]	**< 0.001**
**ATZ**	3.0 ± 0.7[a]	3.8 ± 0.3[b / b]	2.6 ± 0.5[a]	2.7 ± 0.4[a]	2.9 ± 0.5[a]	3.0 ± 0.4[a]	**< 0.001**
**CHL**	3.0 ± 0.7[a]	3.8 ± 0.3[b / b]	2.3 ± 0.2[c]	2.3 ± 0.3[c]	2.5 ± 0.4[c]	2.3 ± 0.3[c]	**< 0.001**
**60 min**							
**NPX**	3.0 ± 0.8[a]	3.8 ± 0.5[b / b]	2.7 ± 0.4[ac]	2.7 ± 0.4[a]	2.5 ± 0.4[ac]	2.3 ± 0.3[c]	**< 0.001**
**DCF**	3.0 ± 0.8[a]	3.8 ± 0.5[b / b]	2.2 ± 0.2[cd]	2.2 ± 0.2[cd]	2.3 ± 0.4[c]	2.0 ± 0.4[d]	**< 0.001**
**SX**	3.0 ± 0.8[a]	3.8 ± 0.5[b / b]	2.7 ± 0.4[ab]	2.6 ± 0.4[bc]	2.2 ± 0.3[cd]	2.1 ± 0.3[d]	**< 0.001**
**ATZ**	3.0 ± 0.8[ac]	3.8 ± 0.5[b / b]	2.5 ± 0.3[a]	2.7 ± 0.3[ac]	2.9 ± 0.5[c]	2.9 ± 0.3[c]	**< 0.001**
**CHL**	3.0 ± 0.8[a]	3.8 ± 0.5[b / b]	2.4 ± 0.3[cd]	2.3 ± 0.3[c]	2.5 ± 0.3[ad]	2.4 ± 0.3[cd]	**< 0.001**
**BCF (Hz)**							
**5 min**							
**NPX**	22.3 ± 3.0	21.1 ± 3.0[a]	21.2 ± 3.2	20.6 ± 3.3	20.5 ± 2.8	18.4 ± 1.9	0.101
**DCF**	22.3 ± 3.0[a]	21.1 ± 3.0[ab / a]	19.6 ± 1.8[bc]	18.6 ± 1.6[cd]	17.3 ± 1.4[de]	17.0 ± 0.9[e]	**< 0.001**
**SX**	22.3 ± 3.0[a]	21.1 ± 3.0[a]	21.1 ± 3.0[a]	20.8 ± 2.7[a]	15.5 ± 1.1[b]	15.0 ± 1.5[b]	**< 0.001**
**ATZ**	22.3 ± 3.0[a]	21.1 ± 3.0[ac / a]	16.6 ± 2.0[b]	18.8 ± 3.1[bc]	18.7 ± 2.6[bc]	19.7 ± 2.4[abc]	**< 0.001**
**CHL**	22.3 ± 3.0[a]	21.1 ± 3.0[a]	15.3 ± 0.9[b]	14.9 ± 0.9[b]	15.3 ± 2.0[b]	14.7 ± 1.1[b]	**< 0.001**
**30 min**							
**NPX**	20.9 ± 2.2[a]	19.4 ± 2.2[ab / ab]	20.0 ± 2.2[ab]	18.8 ± 2.6[ab]	18.4 ± 2.4[ab]	17.4 ± 1.7[b]	**0.017**
**DCF**	20.9 ± 2.2[a]	19.4 ± 2.2[ab / ab]	18.6 ± 1.7[bc]	18.1 ± 1.8[bc]	16.9 ± 1.0[cd]	16.1 ± 1.5[d]	**< 0.001**
**SX**	20.9 ± 2.2[a]	19.4 ± 2.2[a / ab]	19.9 ± 1.5[a]	19.1 ± 2.3[a]	15.3 ± 0.8[b]	14.2 ± 1.1[b]	**< 0.001**
**ATZ**	20.9 ± 2.2[a]	19.4 ± 2.2[ac / ab]	15.6 ± 1.3[b]	17.9 ± 1.8[c]	18.3 ± 2.3[c]	18.4 ± 2.5[c]	**< 0.001**
**CHL**	20.9 ± 2.2[a]	19.4 ± 2.2[a / ab]	15.5 ± 1.1[b]	15.4 ± 1.4[b]	14.9 ± 2.4[b]	14.7 ± 0.9[b]	**< 0.001**
**60 min**							
**NPX**	19.8 ± 2.9[a]	18.2 ± 1.9[ab / b]	18.8 ± 2.4[ab]	17.5 ± 2.6[ab]	18.0 ± 1.6[ab]	16.7 ± 1.7[b]	**0.052**
**DCF**	19.8 ± 2.9[a]	18.2 ± 1.9[ab / b]	18.3 ± 1.9[ab]	17.9 ± 1.4[ab]	16.3 ± 1.2[bc]	15.8 ± 1.4[c]	**< 0.001**
**SX**	19.8 ± 2.9[a]	18.2 ± 1.9[b / b]	18.9 ± 1.3[a]	18.6 ± 2.0[a]	15.1 ± 0.9[c]	14.1 ± 1.1[c]	**< 0.001**
**ATZ**	19.8 ± 2.9[a]	18.2 ± 1.9[a / b]	15.3 ± 1.2[b]	17.9 ± 1.6[a]	17.6 ± 1.9[a]	17.9 ± 2.2[a]	**0.001**

**CHL**	19.8 ± 2.9[a]	18.2 ± 1.9[a / b]	14.8 ± 0.9[b]	14.1 ± 1.3[b]	14.6 ± 1.5[b]	14.4 ± 1.5[b]	**< 0.001**

Note: T1 - T4 is equal to the ascending concentrations of each individual treatment. Values labelled with different letters (a, b, c, d, e) in black were significantly different between concentrations levels for individual treatments (p <0.05). Values in the same column labelled with different letters (a, b, c,) in red were significantly different between time points for individual treatments. *ALH* , amplitude of lateral head displacement; *BCF* , beat cross frequency; *ATZ* , atrazine; *CAP* , capacitation HTF; *CHL* , chlorpyrifos; *DCF* , diclofenac; *LIN* , linearity; *min* , minutes; *NPX* , naproxen; *SD* , standard deviation; *STR* , straightness; *SX* , sulfamethoxazole; *T* , treatment; *VAP* , average path velocity; *VCL* curvilinear velocity; *VSL* , straight-line velocity; *WOB* , wobble.

Table S4: Induced hyperactivation of human spermatozoa after exposure to different concentration levels of naproxen, diclofenac, sulfamethoxazole, atrazine and chlorpyrifos (n=10) (mean ± SD).

	Treatment						ANOVA
	Control	CAP	T1	T2	T3	T4	
**5 min**							
**NPX**	7.7 ± 9.3[a]	16.2 ± 10.2[a]	3.9 ± 4.5[b]	1.5 ± 1.4[b]	1.7 ± 2.1[bc]	0.2 ± 0.6[c]	**<0.001**
**DCF**	7.7 ± 9.3[a]	16.2 ± 10.2[a]	0.1 ± 0.3[b]	0.1 ± 0.2[b]	0.5 ± 0.7[c]	0.0 ± 0.1[b]	**<0.001**
**SX**	7.7 ± 9.3[a]	16.2 ± 10.2[b]	7.4 ± 10.7[a]	3.0 ± 2.6[a]	0.7 ± 1.5[c]	0.0 ± 0.0[c]	**<0.001**
**ATZ**	7.7 ± 9.3[ad]	16.2 ± 10.2[b]	0.9 ± 1.5[c]	1.5 ± 1.7[dc]	7.5 ± 7.3[a]	5.2 ± 5.7[ad]	**<0.001**
**CHL**	7.7 ± 9.3[a]	16.2 ± 10.2[a]	0.8 ± 1.0[b]	1.5 ± 2.6[b]	1.3 ± 1.9[b]	0.9 ± 1.5[b]	**<0.001**
**15 min**							
**NPX**	12.6 ± 10.5[a]	29.7 ± 8.1[b]	3.8 ± 3.4[c]	2.6 ± 2.0[c]	4.5 ± 4.6[c]	0.2 ± 0.5[d]	**<0.001**
**DCF**	12.6 ± 10.5[a]	29.7 ± 8.1[b]	0.6 ± 0.9[c]	0.4 ± 0.8[c]	0.3 ± 0.6[c]	0.6 ± 1.4[c]	**<0.001**
**SX**	12.6 ± 10.5[a]	29.7 ± 8.1[b]	4.2 ± 3.6[c]	5.2 ± 5.6[c]	0.2 ± 0.3[d]	0.1 ± 0.4[d]	**<0.001**
**ATZ**	12.6 ± 10.5[a]	29.7 ± 8.1[b]	0.4 ± 0.7[c]	2.1 ± 2.7[c]	8.1 ± 5.0[ad]	5.9 ± 4.8[d]	**<0.001**
**CHL**	12.6 ± 10.5[a]	29.7 ± 8.1[b]	0.5 ± 1.2[c]	0.0 ± 0.0[c]	1.3 ± 2.8[c]	0.2 ± 0.4[c]	**<0.001**
**30 min**							
**NPX**	11.8 ± 10.7[a]	27.9 ± 8.6[b]	3.6 ± 3.8[c]	3.9 ± 2.9[c]	3.2 ± 4.1[c]	0.6 ± 0.7[d]	**<0.001**
**DCF**	11.8 ± 10.7[a]	27.9 ± 8.6[b]	0.3 ± 0.5[c]	0.7 ± 0.9[c]	0.1 ± 0.3[c]	0.5 ± 1.1[c]	**<0.001**
**SX**	11.8 ± 10.7[a]	27.9 ± 8.6[b]	5.5 ± 4.5[c]	4.5 ± 4.5[c]	0.1 ± 0.3[d]	0.0 ± 0.0[d]	**<0.001**
**ATZ**	11.8 ± 10.7[a]	27.9 ± 8.6[b]	0.3 ± 0.4[c]	2.3 ± 2.6[d]	5.4 ± 5.4[e]	5.7 ± 2.6[ae]	**<0.001**
**CHL**	11.8 ± 10.7[a]	27.9 ± 8.6[b]	0.4 ± 0.6[cd]	0.4 ± 0.8[cd]	1.1 ± 1.8[d]	0.1 ± 0.2[c]	**<0.001**
**45 min**							
**NPX**	10.0 ± 11.5[a]	28.9 ± 13.2[b]	4.4 ± 4.4[ac]	2.7 ± 2.1[ac]	3.0 ± 4.9[c]	0.0 ± 0.0[d]	**<0.001**
**DCF**	10.0 ± 11.5[a]	28.9 ± 13.2[b]	0.5 ± 0.7[c]	0.1 ± 0.2[cd]	0.0 ± 0.2[cd]	0.0 ± 0.0[d]	**<0.001**
**SX**	10.0 ± 11.5[a]	28.9 ± 13.2[b]	5.9 ± 5.3[a]	6.8 ± 10.1[a]	0.6 ± 1.5[c]	0.1 ± 0.2[c]	**<0.001**
**ATZ**	10.0 ± 11.5[a]	28.9 ± 13.2[b]	0.7 ± 1.7[c]	3.5 ± 3.4[ad]	6.2 ± 5.4[a]	2.5 ± 3.7[cd]	**<0.001**
**CHL**	10.0 ± 11.5[a]	28.9 ± 13.2[b]	0.3 ± 0.9[c]	0.0 ± 0.0[c]	0.3 ± 0.7[c]	0.0 ± 0.0[c]	**<0.001**
**60 min**							
**NPX**	12.1 ± 12.3[a]	27.0 ± 13.7[b]	4.5 ± 3.8[ac]	2.8 ± 2.4[c]	2.7 ± 3.0[c]	0.2 ± 0.6[d]	**<0.001**
**DCF**	12.1 ± 12.3[a]	27.0 ± 13.7[a]	0.8 ± 0.8[b]	0.6 ± 0.8[b]	0.9 ± 1.6[b]	0.0 ± 0.0[c]	**<0.001**

**SX**	12.1 ± 12.3[a]	27.0 ± 13.7[b]	4.4 ± 3.7[c]	7.2 ± 10.4[ac]	0.2 ± 0.3[d]	0.0 ± 0.1[d]	**<0.001**
**ATZ**	12.1 ± 12.3[a]	27.0 ± 13.7[b]	0.3 ± 0.7[c]	3.1 ± 2.6[d]	6.0 ± 5.6[ad]	4.2 ± 4.1[d]	**<0.001**
**CHL**	12.1 ± 12.3[a]	27.0 ± 13.7[b]	0.2 ± 0.6[c]	0.0 ± 0.1[c]	0.3 ± 0.7[c]	0.1 ± 0.4[c]	**<0.001**

Note: T1 - T4 is equal to the ascending concentrations of each individual treatment. Values labelled with different letters (a, b, c, d, e) were significantly different between concentrations levels for individual treatments (p <0.05).

ATZ , atrazine; *CAP,* capacitation HTF; *CHL* , chlorpyrifos; *DCF* , diclofenac; *min* , minutes; *NPX* , naproxen; *SD* , standard deviation; *SX* , sulfamethoxazole; *T* , treatment.

Table S5: Average motility and kinematic parameters of human spermatozoa after 5- and 60-minutes exposure to different concentrations of a naproxen, diclofenac, sulfamethoxazole, atrazine and chlorpyrifos mixture (n=10) (mean ± SD).

	Treatment					ANOVA
	**Control**	**CAP**	**MIX 1 (ng/L)**	**MIX 2 (µg/L)**	**MIX 3 (µg/mL)**	
**Prog (%)**						
**5 min**	34.6 ± 16.2	43.7 ± 16.7	33.4 ± 16.5	33.5 ± 16.9	36.5 ± 16.6	0.742
**60 min**	32.3 ± 16.0	41.1 ± 18.7	29.4 ± 14.2	31.2 ± 13.4	32.6 ± 17.1	0.668
**Tot Mot (%)**						
**5 min**	48.3 ± 15.5	53.4 ± 17.1	47.7 ± 17.2	47.0 ± 16.0	49.2 ± 16.1	0.958
**60 min**	42.5 ± 17.3	51.0 ± 18.6	41.7 ± 15.9	41.9 ± 15.0	43.9 ± 16.8	0.816
**Rapid (%)**						
**5 min**	29.4 ± 15.9	39.8 ± 16.9	26.9 ± 16.2	29.6 ± 17.0	31.2 ± 16.1	0.604
**60 min**	27.9 ± 15.2	38.3 ± 18.5	24.8 ± 13.0	27.2 ± 13.5	29.4 ± 16.5	0.466
**Medium (%)**						
**5 min**	9.0 ± 5.0	6.6 ± 3.5	10.8 ± 4.2	7.5 ± 2.8	9.0 ± 4.0	0.262
**60 min**	7.4 ± 3.0	5.5 ± 2.4	8.4 ± 3.8	7.1 ± 2.7	6.6 ± 2.6	0.392
**Slow (%)**						
**5 min**	9.8 ± 4.2	6.9 ± 1.2	10.0 ± 5.1	9.9 ± 4.2	9.0 ± 4.2	0.603
**60 min**	7.3 ± 2.9	7.2 ± 2.7	8.5 ± 3.2	7.5 ± 3.3	7.8 ± 2.7	0.945
**RP (%)**						
**5 min**	11.5 ± 6.6	13.6 ± 5.9	11.0 ± 7.1	11.9 ± 7.6	13.1 ± 8.1	0.956
**60 min**	10.7 ± 4.7	15.3 ± 8.0	11.1 ± 5.9	11.2 ± 6.1	12.6 ± 7.4	0.604
**MP (%)**						
**5 min**	23.1 ± 10.8	30.1 ± 15.2	22.4 ± 11.9	21.6 ± 11.2	26.3 ± 12.8	0.664
**60 min**	21.6 ± 12.2	25.7 ± 15.3	18.3 ± 10.7	20.0 ± 10.4	20.0 ± 12.5	0.831
**NP (%)**						
**5 min**	13.7 ± 5.4	9.7 ± 1.7	14.3 ± 6.1	13.5 ± 4.6	12.7 ± 5.0	0.422
**60 min**	10.2 ± 3.8	9.9 ± 2.9	12.2 ± 4.2	10.6 ± 4.4	11.2 ± 4.2	0.818
**VCL (µm/s)**						
**5 min**	115.4 ± 25.5	137.6 ± 18.3	107.9 ± 19.6	117.2 ± 22.3	122.1 ± 20.0	0.077

**60 min**	120.1 ± 20.2[ab]	139.6 ± 20.9[a]	109.5 ± 14.2[b]	121.6 ± 17.9[ab]	122.9 ± 22.5[ab]	**0.040**
**VAP (µm/s)**						
**5 min**	53.2 ± 6.4	60.0 ± 4.6	52.5 ± 7.2	55.4 ± 6.3	56.9 ± 6.7	0.131
**60 min**	56.9 ± 5.0	63.1 ± 5.8	54.7 ± 5.7	58.1 ± 6.3	58.0 ± 7.4	0.091
**VSL (µm/s)**						
**5 min**	37.8 ± 4.4	41.5 ± 6.8	37.9 ± 6.5	39.0 ± 5.8	40.6 ± 6.1	0.658
**60 min**	40.8 ± 4.1	44.6 ± 6.6	39.1 ± 7.0	41.9 ± 7.2	41.7 ± 7.0	0.559
**STR (%)**						
**5 min**	68.6 ± 4.0	66.9 ± 6.4	69.0 ± 4.7	67.7 ± 3.4	68.7 ± 4.1	0.865
**60 min**	68.8 ± 4.1	68.5 ± 5.9	67.6 ± 4.7	68.6 ± 5.1	68.7 ± 5.3	0.986
**LIN (%)**						
**5 min**	35.4 ± 7.3	31.8 ± 6.7	36.7 ± 5.4	35.0 ± 5.3	35.4 ± 6.0	0.625
**60 min**	35.7 ± 6.4	34.1 ± 6.8	36.2 ± 4.9	35.5 ± 6.1	35.2 ± 6.5	0.982
**WOB (%)**						
**5 min**	49.6 ± 7.1	45.8 ± 5.4	51.1 ± 4.8	50.1 ± 5.8	49.6 ± 5.4	0.445
**60 min**	50.0 ± 5.9	47.9 ± 6.1	52.0 ± 4.7	50.1 ± 5.5	49.8 ± 5.7	0.717
**ALH (µm)**						
**5 min**	3.4 ± 0.8	4.1 ± 0.6	3.2 ± 0.6	3.5 ± 0.7	3.6 ± 0.6	0.088
**60 min**	3.6 ± 0.7	4.1 ± 0.7	3.3 ± 0.4	3.6 ± 0.6	3.7 ± 0.7	0.086
**BCF (Hz)**						
**5 min**	18.7 ± 1.7	17.8 ± 1.4	18.8 ± 1.6	18.5 ± 1.0	18.4 ± 1.1	0.699
**60 min**	18.0 ± 1.3	18.1 ± 1.4	18.1 ± 1.6	17.9 ± 1.3	17.8 ± 1.3	0.983

Note: MIX 1 - 3 is equal to the ascending concentrations of each individual chemicals inside each collective mixture. Values labelled with different letters (a, b, c, d, e) in black were significantly different between mixture concentration levels (p <0.05). *ALH* , amplitude of lateral head displacement; *BCF* , beat cross frequency; *CAP* , capacitation HTF; *LIN* , linearity; *min* , minutes; *MIX* , collective mixture; *SD* , standard deviation; *STR* , straightness; *VAP* , average path velocity; *VCL* , curvilinear velocity; *VSL* , straight-line velocity; *WOB* , wobble.
